# Streptococcus suis MsmK: Novel Cell Division Protein Interacting with FtsZ and Maintaining Cell Shape

**DOI:** 10.1128/mSphere.00119-21

**Published:** 2021-03-17

**Authors:** Mei-Fang Tan, Qiao Hu, Zhe Hu, Chun-Yan Zhang, Wan-Quan Liu, Ting Gao, Liang-Sheng Zhang, Lun Yao, Hai-Qin Li, Yan-Bin Zeng, Rui Zhou

**Affiliations:** a Institute of Animal Husbandry and Veterinary Science, Jiangxi Academy of Agricultural Sciences, Nanchang, China; b State Key Laboratory of Agricultural Microbiology, College of Veterinary Medicine, Huazhong Agricultural University, Wuhan, China; c Institute of Animal Husbandry and Veterinary Science, Hubei Academy of Agricultural Sciences, Wuhan, China; d Cooperative Innovation Center of Sustainable Pig Production, Wuhan, China; e International Research Center for Animal Disease (Ministry of Science & Technology of China), Wuhan, China; University of Iowa

**Keywords:** *Streptococcus suis*, MsmK, FtsZ, cell shape, peripheral cell wall synthesis

## Abstract

Bacteria of different shapes have adopted distinct mechanisms to faithfully coordinate morphogenesis and segregate their chromosomes prior to cell division. Despite recent focuses and advances, the mechanism of cell division in ovococci remains largely unknown. Streptococcus suis, a major zoonotic pathogen that causes problems in human health and in the global swine industry, is an elongated and ellipsoid bacterium that undergoes successive parallel splitting perpendicular to its long axis. Studies on cell cycle processes in this bacterium are limited. Here, we report that MsmK (multiple sugar metabolism protein K), an ATPase that contributes to the transport of multiple carbohydrates, has a novel role as a cell division protein in S. suis. MsmK can display ATPase and GTPase activities, interact with FtsZ via the N terminus of MsmK, and promote the bundling of FtsZ protofilaments in a GTP-dependent manner *in vitro*. Deletion of the C-terminal region or the Walker A or B motif affects the affinity between MsmK and FtsZ and decreases the ability of MsmK to promote FtsZ protofilament bundling. MsmK can form a complex with FtsZ *in vivo*, and its absence is not lethal but results in long chains and short, occasionally anuclear daughter cells. Superresolution microscopy revealed that the lack of MsmK in cells leads to normal septal peptidoglycan walls in mother cells but disturbed cell elongation and peripheral peptidoglycan synthesis. In summary, MsmK is a novel cell division protein that maintains cell shape and is involved in the synthesis of the peripheral cell wall.

**IMPORTANCE** Bacterial cell division is a highly ordered process regulated in time and space and is a potential target for the development of antimicrobial drugs. Bacteria of distinct shapes depend on different cell division mechanisms, but the mechanisms used by ovococci remain largely unknown. Here, we focused on the zoonotic pathogen Streptococcus suis and identified a novel cell division protein named MsmK, which acts as an ATPase of the ATP-binding cassette-type carbohydrate transport system. MsmK has GTPase and ATPase activities. *In vitro* protein assays showed that MsmK interacts with FtsZ and promotes FtsZ protofilament bundling that relies on GTP. Superresolution microscopy revealed that MsmK maintains cell shape and is involved in peripheral peptidoglycan synthesis. Knowledge of the multiple functions of MsmK may broaden our understanding of known cell division processes. Further studies in this area will elucidate how bacteria can faithfully and continually multiply in a constantly changing environment.

## INTRODUCTION

Bacteria exhibit various morphologies and sizes, from rod-shaped species to spherical cells, thereby illustrating different mechanisms that guide faithful cell growth and division processes (1). Well-coordinated events, including cell wall synthesis, chromosome organization and segregation, correct division site selection, and cytokinesis, are the fundamental aspects of generating two identical daughter cells in nearly all living bacteria (2). Compared with those of rod-shaped (e.g., Bacillus subtilis and Escherichia coli) or spherical (e.g., Staphylococcus aureus) microorganisms, the molecular basis underlying morphogenesis in ovococci is poorly studied (1). Research on cell cycle processes in ovococci is based on the best-known ovoid bacterium, Streptococcus pneumoniae, which serves as the model bacterium to study fundamental morphogenetic questions (3–5).

Peptidoglycan (PG) is the major constituent of the bacterial cell wall (6), and cell shape is maintained by its flexible sacculus (1). Ovococci are elongated ellipsoids and are divided in successive parallel planes that are perpendicular to their long axis (7). Their cell cycle begins with the assembly of the highly conserved tubulin-like protein FtsZ (filamentous temperature-sensitive protein Z) at midcell (8). The ring composed of FtsZ protofilaments provides a scaffold for the binding of dozens of cell division proteins, including stabilizers, scaffolders, shapers, regulators, and penicillin-binding proteins (PBPs), that synthesize nascent PG and catalyze transglycosylation and transpeptidation reactions (9). Unlike that in spherical cocci, peripheral and septal cell wall synthesis is required in ovococci during cell division for elongation and synthesis of the division septum, respectively (10). Peripheral cell wall synthesis leads to the slight longitudinal elongation of ovococci in close proximity to the division site, thereby inserting material between the future equatorial rings and the present division site (1). According to the two-state model for the two cell wall synthesis machineries of ovococci, peripheral synthesis occurs in a broad band at the midcell but not along the lateral wall (1). If septal growth is inhibited, then ovococci can assume a rod shape; if only peripheral growth is inhibited, then ovococci can become round cells (7). Despite recent advances, the mechanisms of cell division in ovococci, specifically that balancing the two types of cell wall growth and the complete list of cytoskeletal elements that maintain cell shape, remain unknown.

Streptococcus suis is a major zoonotic pathogen that affects humans and swine worldwide (11). As a primary causative pathogen, S. suis has been reported in more than 30 countries and has claimed at least 1,600 human lives worldwide (12). Potent inhibitors and therapeutic agents to control streptococcal infections are urgently needed to tackle the rapidly increasing number of antibiotic-resistant strains among clinical isolates (13). Bacterial cell division is an attractive target for the development of such drugs (14). Similar to S. pneumoniae, S. suis can be observed as isolated cells, diplococci, or small chains shaped like elongated ellipsoids. Although it possesses the same cell division pattern, the cell division process in S. suis probably differs in certain details from that of S. pneumoniae due to its distinct colonization characteristics, pathogenic habitats, and cell wall gene cluster organization and distribution (3).

Here, we aimed to identify novel cell division proteins in S. suis by using bioinformatics technology, and we report the participation of MsmK (multiple sugar metabolism protein K) in cell division for the first time. As a component of the carbohydrate transport system, MsmK binds to FtsZ *in vitro* and *in vivo*. MsmK can promote FtsZ protofilament bundling *in vitro*. The deletion of MsmK in S. suis increases the number of cells in the chain and leads to abnormal cell morphology. On the basis of observation of PG synthesis pattern, MsmK is a novel element that maintains cell shape and is involved in the synthesis of the peripheral cell wall.

## RESULTS

### MsmK is an FtsZ-interacting protein.

**FIG 1:**
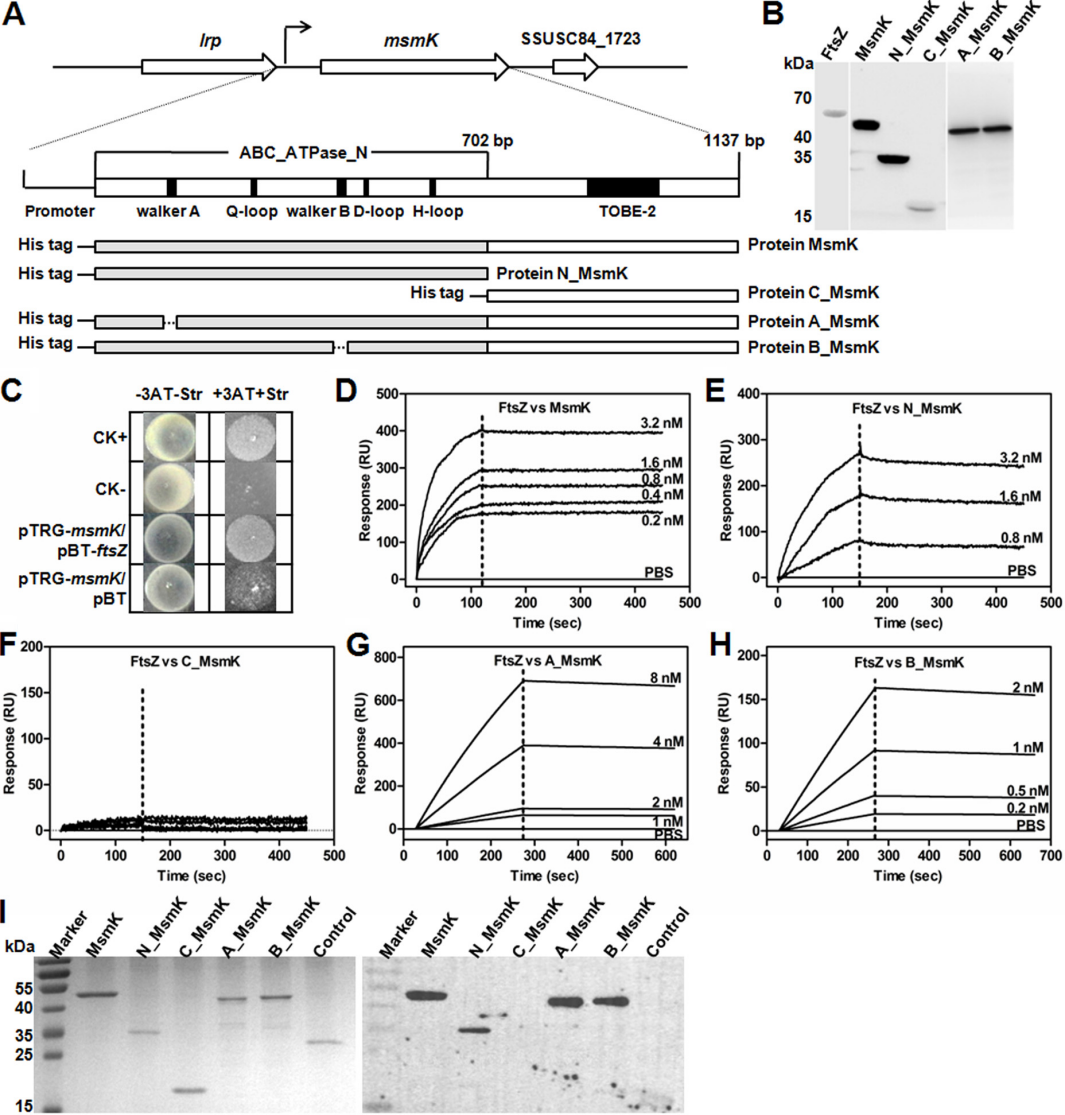
MsmK binds to FtsZ via its N terminus. (A) Schematic of *msmK* locus, gene domain organization, and descriptions of His tag-fused protein constructs. MsmK is encoded by a single open reading frame of 1,137 bp and produces an ATPase protein with 378 amino acid residues. Located upstream of the *msmK* locus is a gene for a leucine-rich protein (*lrp*). Downstream of the *msmK* locus is a putative membrane protein. The gray bars represent the N-terminal domain of MsmK. The white bars represent the C-terminal domain of MsmK. The dotted lines represent the amino acid sequences of the motifs that have been deleted in various protein mutants. (B) Western blot assay for the characterization of the His tag-fused proteins. Purified proteins were probed with anti-His monoclonal antibodies. (C) Bacterial two-hybrid assay of the MsmK-FtsZ interaction. pBT- and pTRG-related plasmids were cotransformed into the reporter strain, and positive cotransformants were selected. The liquid culture of each cotransformant was spotted onto selective (+3AT+Str; medium with 3-amino-1,2,4-triazole [3-AT] and streptomycin) and nonselective (−3AT-Str; medium without 3-AT and streptomycin) plates. CK+, a cotransformant containing pBT-LGF2 and pTRG-Gal as a positive control; CK−, a cotransformant containing pBT and pTRG as a negative control. A cotransformant containing pTRG-*msmK* and pBT was the self-activation control. (D to H) SPR assays of the interaction between FtsZ and MsmK (D), N_MsmK (E), C_MsmK (F), A_MsmK (G), and B_MsmK (H). His-tagged FtsZ was immobilized onto sensor chips. Buffer without protein was used as a negative control in all SPR assays. (D) Full-length MsmK was diluted into a series of concentration gradients and was used as the analyte. *K_a_*, 1.00 × 10^3^ 1/Ms; *K_d_*, 1.00 × 10^−5^ 1/s; *K_D_*, 1.00 × 10^−8^ M. “1/Ms” and “1/s” are units of the rate constants. (E) The N terminus of MsmK was diluted into three concentration gradients and was used as the analyte. *K_a_*, 1.00 × 10^3^ 1/Ms; *K_d_*, 3.32 × 10^−4^ 1/s; *K_D_*, 3.32 × 10^−7^ M. (F) The C terminus of MsmK was diluted to 0, 0.4, 0.8, 1.6, and 3.2 nM. (G) A Walker A deletion mutant of MsmK was diluted into a series of concentration gradients and was used as the analyte. *K_a_*, 2.51 × 10^5^ 1/Ms; *K_d_*, 1.02 × 10^−4^ 1/s; *K_D_*, 4.06 × 10^−10^ M. (H) A Walker B deletion mutant of MsmK was diluted into a series of concentration gradients and was used as the analyte. *K_a_*, 4.27 × 10^5^ 1/Ms; *K_d_*, 1.32 × 10^−4^ 1/s; *K_D_*, 3.09 × 10^−10^ M. (I) Far-Western blot analysis of MsmK and its derivatives incubated with FtsZ. An ATPase (SSUSC84_1444), the homologous protein of MsmK with high identity (40.4%) in S. suis, served as the negative control.

A protein-protein interaction subnetwork in cell division of S. suis was constructed through homologous protein mapping. Predicted results showed that MsmK, an ATPase belonging to the ATP-binding cassette (ABC)-type sugar transport system, contributes to multiple-carbohydrate utilization (15) and potentially interacts with the most highly conserved divisome component, FtsZ. Conserved-domain analysis showed that the N-terminal region of MsmK contains the characteristic motifs of an ATPase (Fig. 1A). Walker A and B motifs are associated with ATP binding and hydrolysis, respectively (16). For follow-up protein experiments, His-tagged MsmK protein and its derivate proteins, N_MsmK (amino acids [aa] 1 to 234), C_MsmK (aa 235 to 378), A_MsmK, and B_MsmK were expressed and purified (Fig. 1A). A_MsmK and B_MsmK are the protein mutants generated upon deletion of the Walker A (GPSGCGKS; aa 40 to 47) and B (VFLMD; aa 158 to 162) motifs, respectively. The authenticity of the purified proteins was checked using an anti-His monoclonal antibody and Western blotting (Fig. 1B).

A bacterial two-hybrid assay was conducted. The fusion strains carrying full-length *msmK* and *ftsZ* grew well on the screening medium, whereas the negative control and the self-activation control did not grow (Fig. 1C). The finding confirmed the interaction of MsmK and FtsZ. Surface plasmon resonance (SPR) was then used to investigate the specificity of MsmK-FtsZ interaction *in vitro*. After conditioning and activation of sensor chips, His-FtsZ was quantitatively assembled on the chip surface as the ligand. Injecting full-length MsmK (Fig. 1D) or its N-terminal domain (Fig. 1E) produced a response proportional to the injected amount. No such signal was obtained when the C-terminal domain (Fig. 1F) or buffer instead of proteins was injected. At the same binding constant (*K_a_*), the dissociation constant (*K_d_*) or equilibrium dissociation constant (*K*_D_) of the MsmK-FtsZ complex is lower than that obtained from the N_MsmK-FtsZ complex. This result revealed a high-affinity interaction between MsmK and FtsZ and a weak binding of N_MsmK to FtsZ.

The associations of FtsZ-A_MsmK (Fig. 1G) and FtsZ-B_MsmK (Fig. 1H) were subsequently tested. When two mutant proteins were used as analytes, the binding abilities of FtsZ-A_MsmK and FtsZ-B_MsmK were higher than that of MsmK-FtsZ. According to calculated *K_D_* values, the affinity among the four FtsZ-interacting MsmK constructs was in the order A_MsmK > B_MsmK > MsmK > N_MsmK (Fig. 1D to H). This result indicated that the deletion of the Walker A or B motif did not abolish but increased the affinities of MsmK and FtsZ. MsmK and its derivatives were further subjected to far-Western blot analysis to investigate their binding properties to FtsZ. FtsZ bound to MsmK, N_MsmK, A_MsmK, and B_MsmK but not to C_MsmK (Fig. 1I). These observations strongly support the direct interaction of MsmK with FtsZ via its N terminus *in vitro*.

### Dual ATPase and GTPase activity of MsmK.

**FIG 2:**
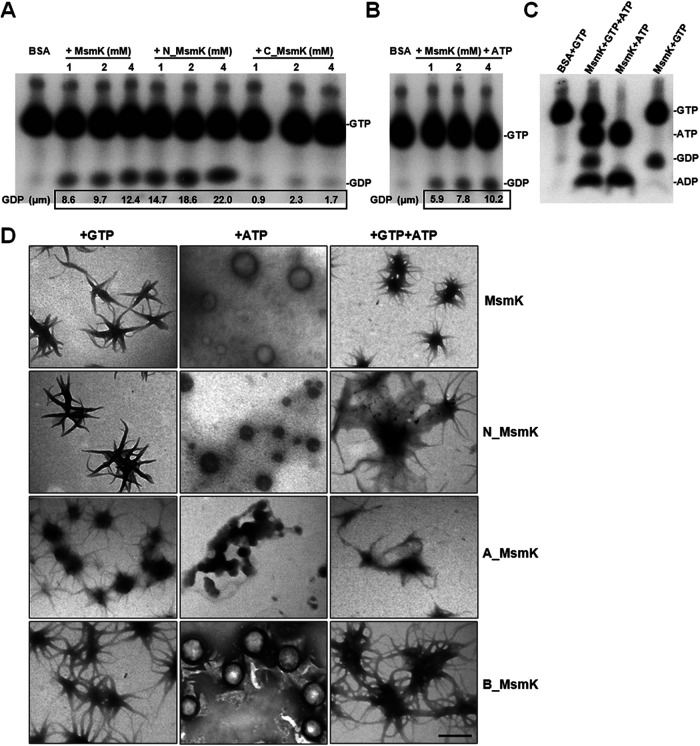
GTPase and ATPase activities of MsmK. (A) Thin-layer chromatography (TLC) analysis of the GTPase activity of MsmK constructs. Purified MsmK, N_MsmK, and C_MsmK proteins were assayed to determine the GTPase activity in the presence of [α-^32^P]GTP supplemented with GTP to a final concentration of 2 mM. Reaction mixtures were analyzed by TLC and autoradiography. (B) TLC analysis of the GTPase activity of MsmK with ATP. MsmK was assayed to determine the GTPase activity in the presence of [α-^32^P]GTP (up to 2 mM) and 1 mM ATP. (C) TLC analysis of the GTPase and ATPase activities of MsmK. MsmK at 2 mM was assayed to determine the bifunctional activities in the presence of [α-^32^P]GTP (up to 2 mM) and/or [α-^32^P]ATP (up to 1 mM). For all GTPase assays, albumin bovine serum (BSA) was used as a negative control. Identical reaction conditions were ensured by compensating for various amounts of protein with a storage buffer in all cases. The amount of [α-^32^P]GDP was quantified using ImageJ2x and was used to calculate the overall amount of GDP generated in the reactions. (D) Electron-microscopic examination of MsmK and its derivatives (0.6 μM each) in the presence of 2 mM GTP and/or 1 mM ATP. Bar, 0.5 μm.

The MsmK protein has ATP binding and ATP hydrolysis activities (15). Here, we found that MsmK could hydrolyze GTP in a concentration-dependent manner (Fig. 2A). The amount of free GDP released from the substrate GTP increased in the presence of 1, 2, and 4 μM MsmK. Equivalent experiments on N_MsmK presented similar tendencies toward increases in GDP release compared with the full-length MsmK. Equal quantities of C_MsmK or bovine serum albumin did not show hydrolysis behavior. After the addition of ATP to the GTP enzyme reaction mixtures, the GTP hydrolysis efficiency of MsmK was decreased (Fig. 2B) compared with that of the reaction system without ATP. ^32^P-labeled GTP and/or ^32^P-labeled ATP were simultaneously incubated with MsmK, and the results confirmed that MsmK could display ATPase and GTPase activities (Fig. 2C).

The polymerization characteristics of MsmK and its derivatives in the presence of K^+^, Mg^2+^, and ATP, and/or GTP were examined by transmission electron microscopy (TEM). As shown in Fig. 2D, the incubation of MsmK with GTP resulted in the formation of regular short slices that spontaneously gathered together. The combination of MsmK with ATP polymerized into spherical particles with various diameters. MsmK could form the third sea urchin-like form in the presence of GTP and ATP. No distinct structure was found when MsmK was incubated in buffer without GTP or ATP (data not shown). These observations indicated that MsmK can form distinct polymers in an ATP- and/or GTP-dependent manner. Moreover, N_MsmK and B_MsmK showed polymerization patterns roughly similar to that of full-length MsmK in the presence of GTP, ATP, or GTP and ATP. Incubating A_MsmK with GTP still produced sea urchin-like polymers, but the ability of MsmK to form spherical polymers with ATP was severely disrupted by the deletion of the Walker A motif.

### MsmK promotes FtsZ protofilament bundling *in vitro*.

**FIG 3:**
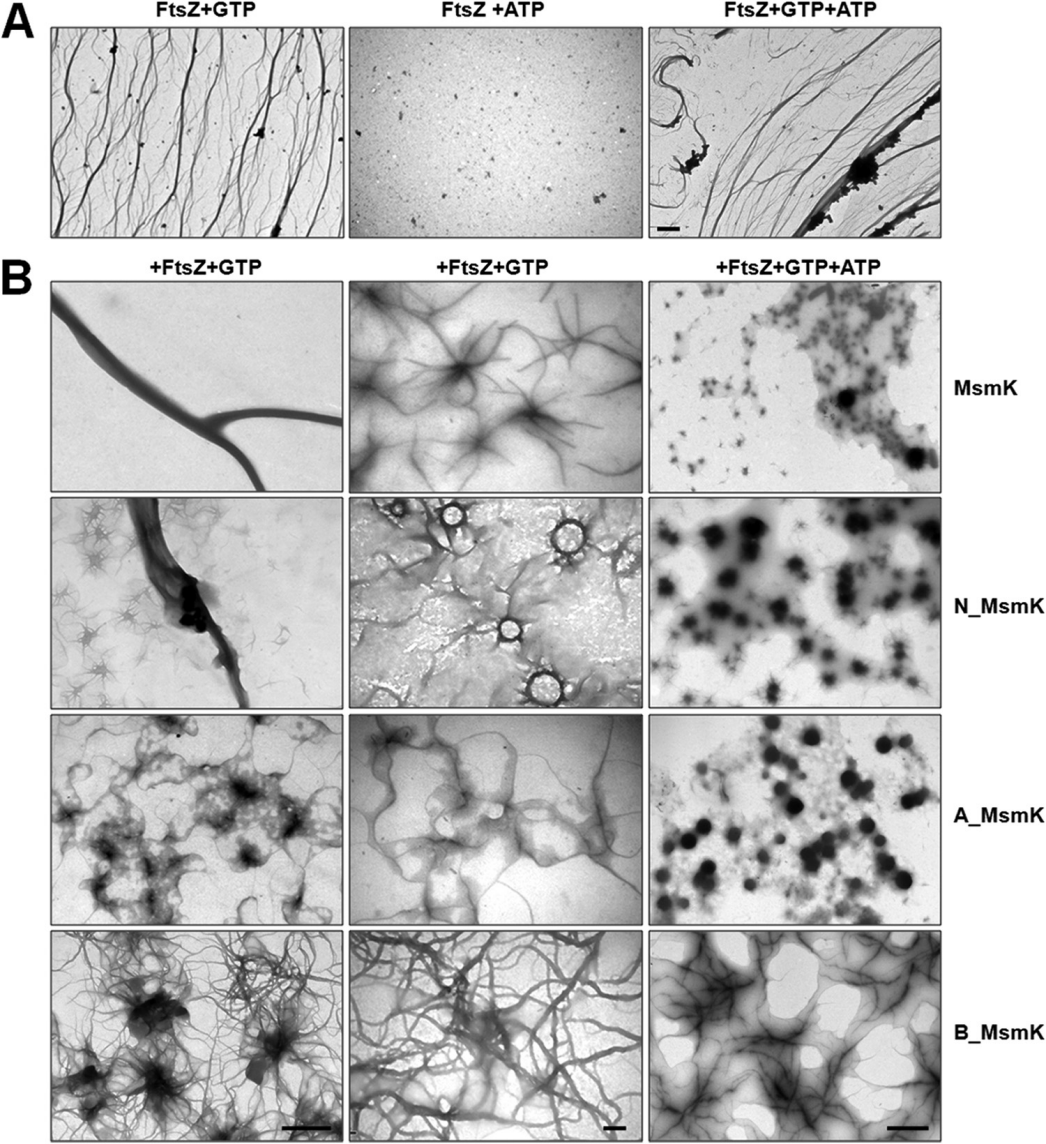
MsmK enhanced the bundle of FtsZ polymers *in vitro*. (A) FtsZ (0.6 μM) was incubated with 2 mM GTP and/or 1 mM ATP. Bar, 2 μm. (B) Electron microscopic examination of the products of polymerization reactions. A sample of 0.6 μM FtsZ was incubated with 0.6 μM MsmK, N_MsmK, A_MsmK, or B_MsmK. Reactions were started by adding 2 mM GTP and/or 1 mM ATP. Bars, 1 μm (left and right) and 200 nm (middle).

TEM was performed to investigate the morphological changes of FtsZ during protofilament formation and bundling. FtsZ assembled into an extensive network of long and thin polymers in the presence of GTP but not ATP (Fig. 3A). As shown in Fig. 3B, incubating FtsZ and MsmK with GTP resulted in the formation of thick filaments with diameters of approximately 300 nm. We observed that the scattered FtsZ protofilaments were connected by a central point, which likely corresponds to the polymerization mode of the two proteins. The mixture of N_MsmK and FtsZ with GTP could also induce protofilament bundling. Interestingly, a large number of clusters with attached FtsZ protofilaments were observed. C_MsmK showed no effect on FtsZ polymerization (see Fig. S2 in the supplemental material). However, the addition of A_MsmK or B_MsmK under the same conditions reduced the FtsZ bundling, especially in the case of A_MsmK. A large number of protein masses were attached to FtsZ protofilaments when FtsZ was incubated with A_MsmK in the presence of GTP. Also, the addition of extra ATP in mixtures negatively affected the formation of the above structures.

10.1128/mSphere.00119-21.5 FIG S2 Electron microscopic examination of the C_MsmK and FtsZ polymerization reaction. A sample of 0.6 μM FtsZ was incubated with 0.6 μM C_MsmK. Reactions were started by adding 2 mM GTP. Download FIG S2, TIF file, 0.2 MB. Copyright © 2021 Tan et al. 2021 Tan et al. https://creativecommons.org/licenses/by/4.0/ This content is distributed under the terms of the Creative Commons Attribution 4.0 International license.

**FIG 4:**
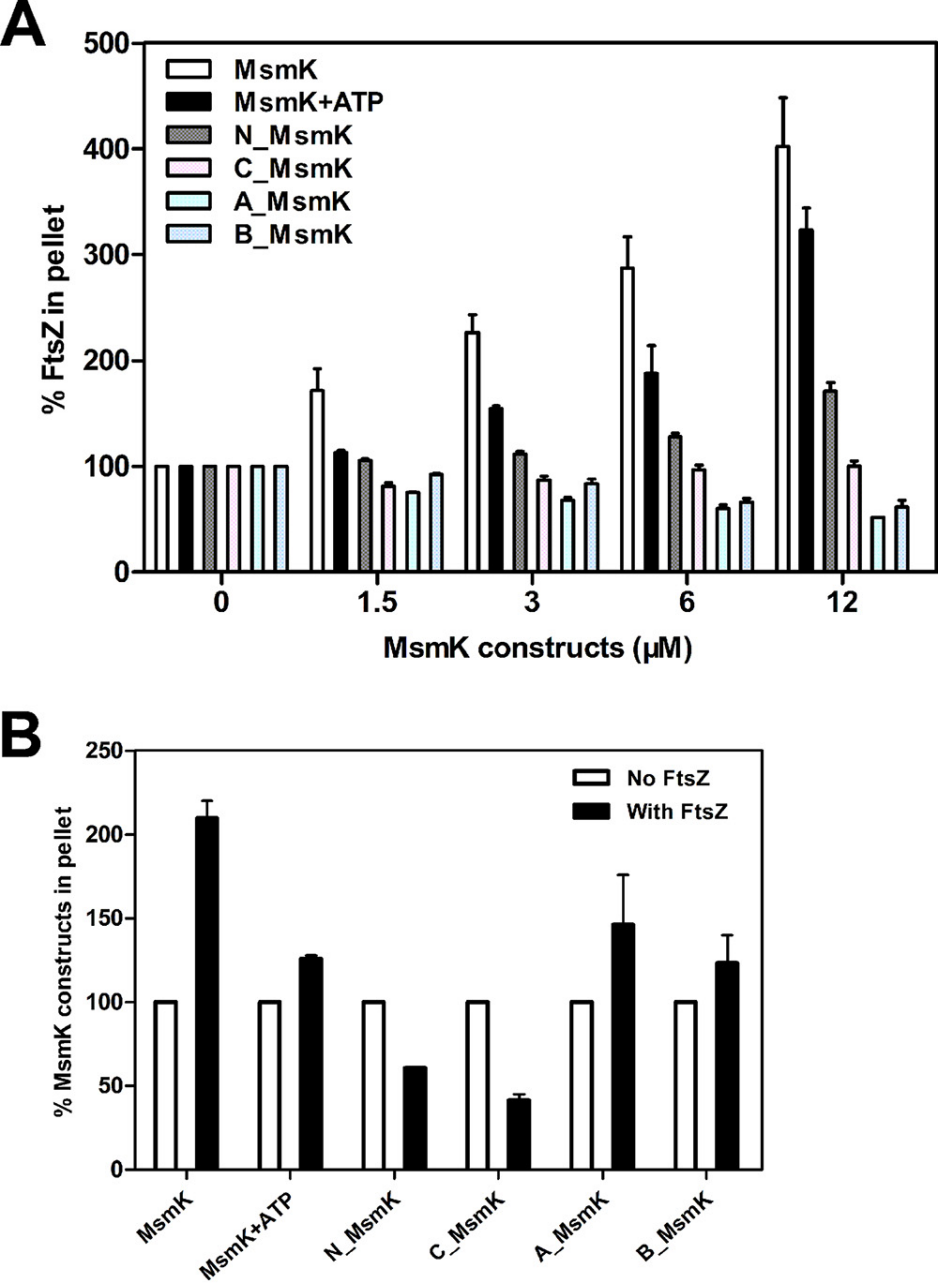
Cosedimentation of FtsZ and MsmK polymers *in vitro*. FtsZ (6 μM) was incubated with increasing amounts of MsmK constructs in the presence of 2 mM GTP. If needed, 1 mM ATP was added. Mixtures were then subjected to high-speed centrifugation. Equivalent aliquots of pellet fractions were analyzed by Coomassie blue staining of electrophoresis gels (Fig. S1). The amounts of specific proteins in pellet were analyzed from three independent experiments using ImageJ2x. (A) Sedimentation of FtsZ in the presence of various MsmK constructs. The amounts of pelleted FtsZ in the absence of MsmK or its derivatives are shown as percentages. (B) Sedimentation of MsmK from pellet fractions with or without FtsZ. The amounts of pellet MsmK constructs in the absence of FtsZ are shown as percentages.

Ultracentrifugation was used to sediment FtsZ protofilaments and any bound proteins (Fig. 4A). The addition of increasing MsmK amounts continuously increased FtsZ sedimentation, indicating that FtsZ polymerization is positively promoted by MsmK. Incorporating ATP marginally reduced the amount of FtsZ in pellets compared with that in the mixtures containing MsmK, FtsZ, and GTP. Sediments with low efficiency of FtsZ polymer formation were observed in the presence of N_MsmK. In contrast, A_MsmK and B_MsmK inhibited FtsZ polymerization. These results confirmed TEM findings. In addition, the MsmK constructs recovered in the pellet fraction were qualitatively analyzed (Fig. 4B). Under the same conditions, the cosedimentation proportion of MsmK constructs was in the order MsmK > A_MsmK > MsmK with ATP > B_MsmK > N_MsmK > C_MsmK.

10.1128/mSphere.00119-21.4 FIG S1 Results of sedimentation assays. Diverse sedimentation results of FtsZ in the presence of MsmK (A), MsmK and ATP (B), N_MsmK (C), C_MsmK (D), A_MsmK (E), and B_MsmK (F). Lane M represents molecular weight markers. Polymerization was induced by addition of 2 mM GTP. If needed, 1 mM ATP was added. Polymers were recovered through ultracentrifugation and analyzed by Coomassie blue staining of electrophoresis gels. Download FIG S1, TIF file, 2.1 MB. Copyright © 2021 Tan et al. 2021 Tan et al. https://creativecommons.org/licenses/by/4.0/ This content is distributed under the terms of the Creative Commons Attribution 4.0 International license.

### Formation of the MsmK-FtsZ complex *in vivo*.

**FIG 5:**
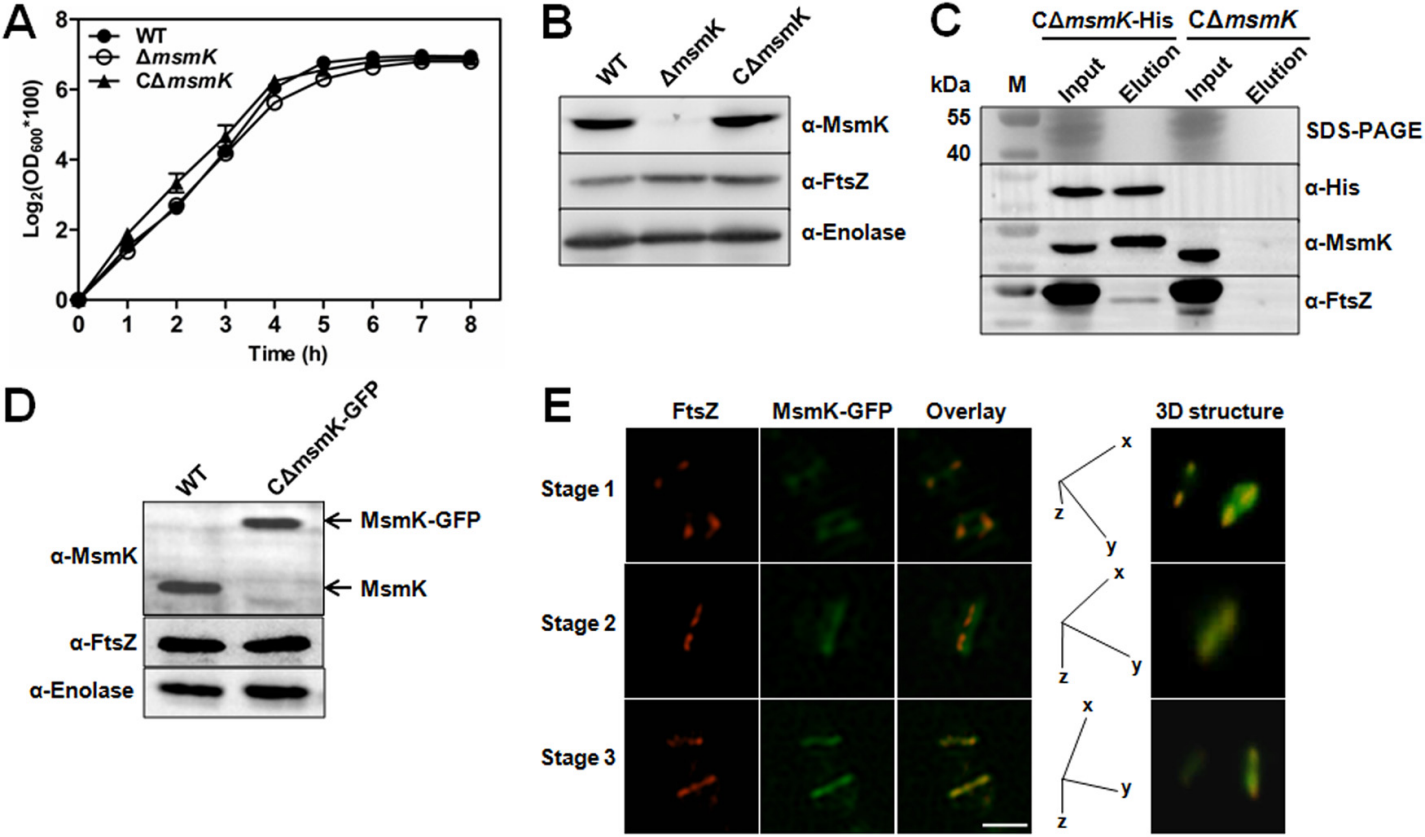
MsmK interacts with FtsZ *in vivo*. (A) Growth characteristics of S. suis strains. Strains were grown in TSB medium at 37°C. The turbidity (OD_600_) of the cultures was measured at 1-h intervals. (B) Identification of S. suis strains. The supernatant of bacterial lysis was separated using SDS-PAGE and probed with mouse anti-MsmK, anti-FtsZ, or anti-enolase serum. (C) Immunoprecipitation to determine the complexes of MsmK and FtsZ *in vivo*. Formaldehyde-treated bacteria were ultrasonically broken, and supernatants were separated using SDS-PAGE. Bait or prey protein was probed with the anti-His antibody, anti-MsmK serum, or anti-FtsZ serum. Lane M represents molecular weight markers. (D) Detection of the expression of MsmK-GFP *in vivo*. Targeted proteins were probed with the anti-MsmK, anti-FtsZ, or anti-enolase serum. (E) Subcellular localization of FtsZ (red) and MsmK-GFP (green) in S. suis. CΔ*msmK-*GFP cells were stained with the anti-FtsZ mouse serum and Cy3-labeled anti-mouse IgG. Overlays indicated the colocalization of FtsZ and MsmK-GFP. *z*-axis images were captured by rotating a section of the midcell region around the *x* or the *y* axis and reconstructed for three-dimensional structure images. Calibration: *x*, 0.03 μm; *y*, 0.03 μm; *z*, 0.20 μm. Bar, 2 μm.

Wild-type (WT) S. suis strain SC19 (serotype 2) was isolated from a diseased pig during an epidemic outbreak in 2005 in Sichuan, China (17). The erythromycin marker mutant (Δ*msmK* strain) was previously prepared (15). The E. coli-S. suis shuttle vector pSET2 carrying the *msmK* expression cassette under the control of its native promoter was transformed into the Δ*msmK* strain to construct the strain CΔ*msmK* for functional complementation (15). During the growth experiment, the Δ*msmK* strain showed a growth trend similar to that of the WT strain, although the values of the optical density at 600 nm (OD_600_) slightly decreased at the late logarithmic phase (Fig. 5A). The protein expression level of FtsZ in the mutant was not affected, as revealed by Western blot analysis (Fig. 5B).

pSET2 carrying the *msmK* expression cassette with a coding sequence for a His tag at the C terminus was constructed and electroporated into the Δ*msmK* strain, which produced the strain CΔ*msmK*-His, to study *in vivo* complexes containing MsmK and FtsZ. Exponentially growing cells of CΔ*msmK*-His were cross-linked using formaldehyde and then subjected to immunoprecipitation experiments on nickel-nitrilotriacetic acid (Ni-NTA) resins. The input mixed bacterial proteins and the final eluted protein fractions were detected by Western blotting analysis by using antibodies to His tag, MsmK, and FtsZ. Control mock pulldowns were run on cross-linked cells of CΔ*msmK* expressing MsmK instead of the His-tagged MsmK and were used as the background control. The prey protein, endogenous FtsZ of S. suis, was attached to the bait protein (Fig. 5C), showing that MsmK could form complexes with FtsZ *in vivo*.

The subcellular localization of MsmK and FtsZ in S. suis cells was examined using immunofluorescence microscopy. The CΔ*msmK*-GFP strain, which expressed MsmK with green fluorescent protein (GFP) at the C terminus (Fig. 5D), was probed using the FtsZ-specific antibody and the Cy3-conjugated anti-mouse IgG. Cells at different division stages (18) were labeled (designated stages 1 to 3). The superresolution images obtained are arranged sequentially in Fig. 5E. Three-dimensional microscopy confirmed that MsmK-GFP colocalized with the ring of FtsZ throughout the cell cycle, revealing that MsmK is a FtsZ-interacting partner and localizes to the site of division.

### Absence of MsmK results in the formation of chains and short cells.

**FIG 6:**
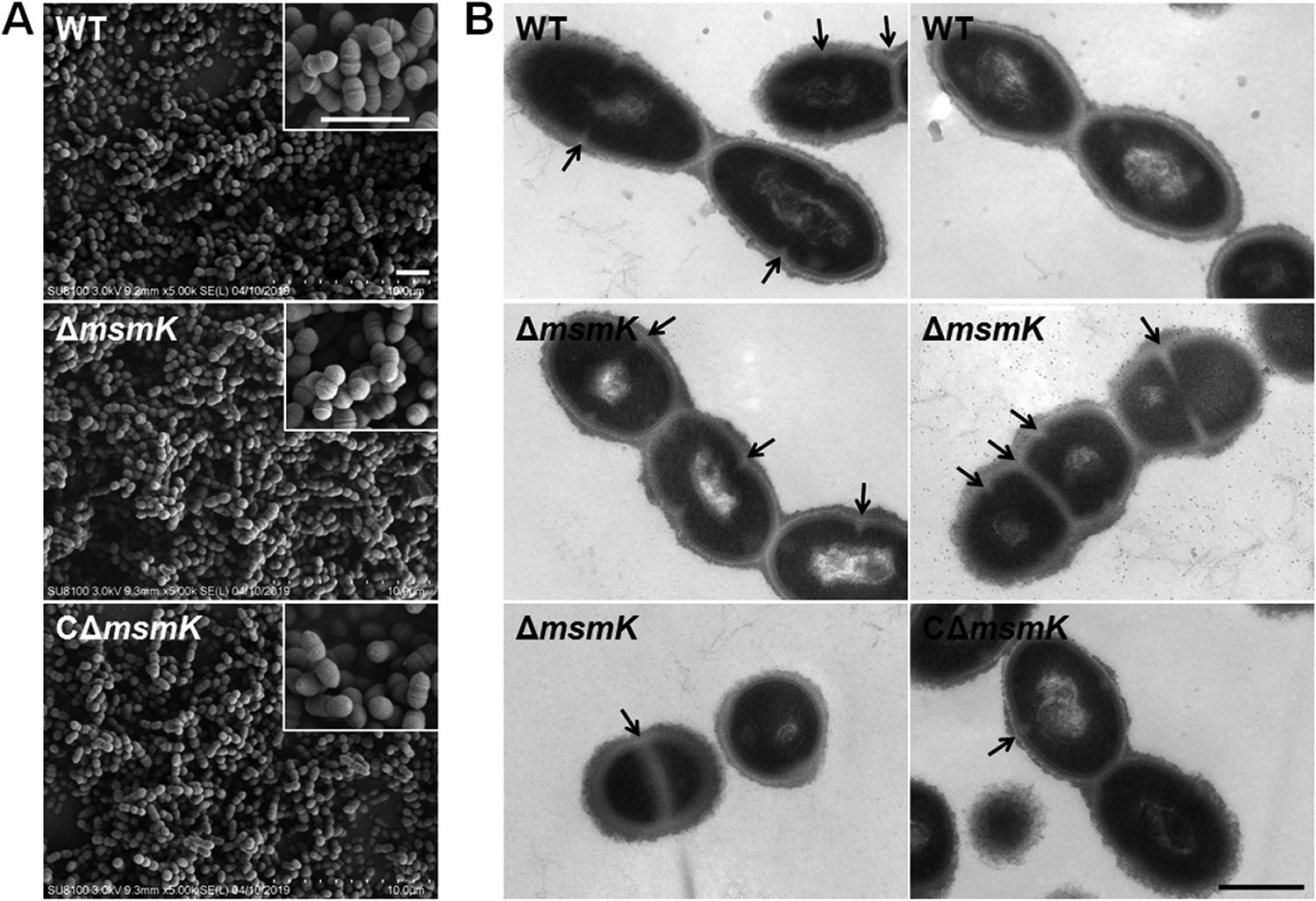
Bacterial morphology characterization of S. suis strains. (A) Scanning electron micrographs of the WT, mutant, and complemented strains. Bars, 2 μm. (B) Transmission electron micrographs of the WT, mutant, and complemented strains. Arrows indicate newborn cellular septum formation sites. Bar, 500 nm.

Gram-staining test showed that MsmK absence lengthens the bacterial chains (19). *In vitro* and *in vivo* protein assays indicated that MsmK is linked to cell division. Scanning electron microscopy (SEM) (Fig. 6A) and TEM (Fig. 6B) were accordingly performed to examine the morphology of the S. suis strains. SEM confirmed the Gram-staining findings. More chains were present in the field of view for the mutant cells than the WT cells (Fig. 6A). Analysis of TEM images showed that chains (at least three well-structured cells) were formed in 4.8% (14/290) and 28.9% (108/374) of the WT and mutant cells, respectively, suggesting the low degree of cell separation of the mutant cells.

**FIG 7:**
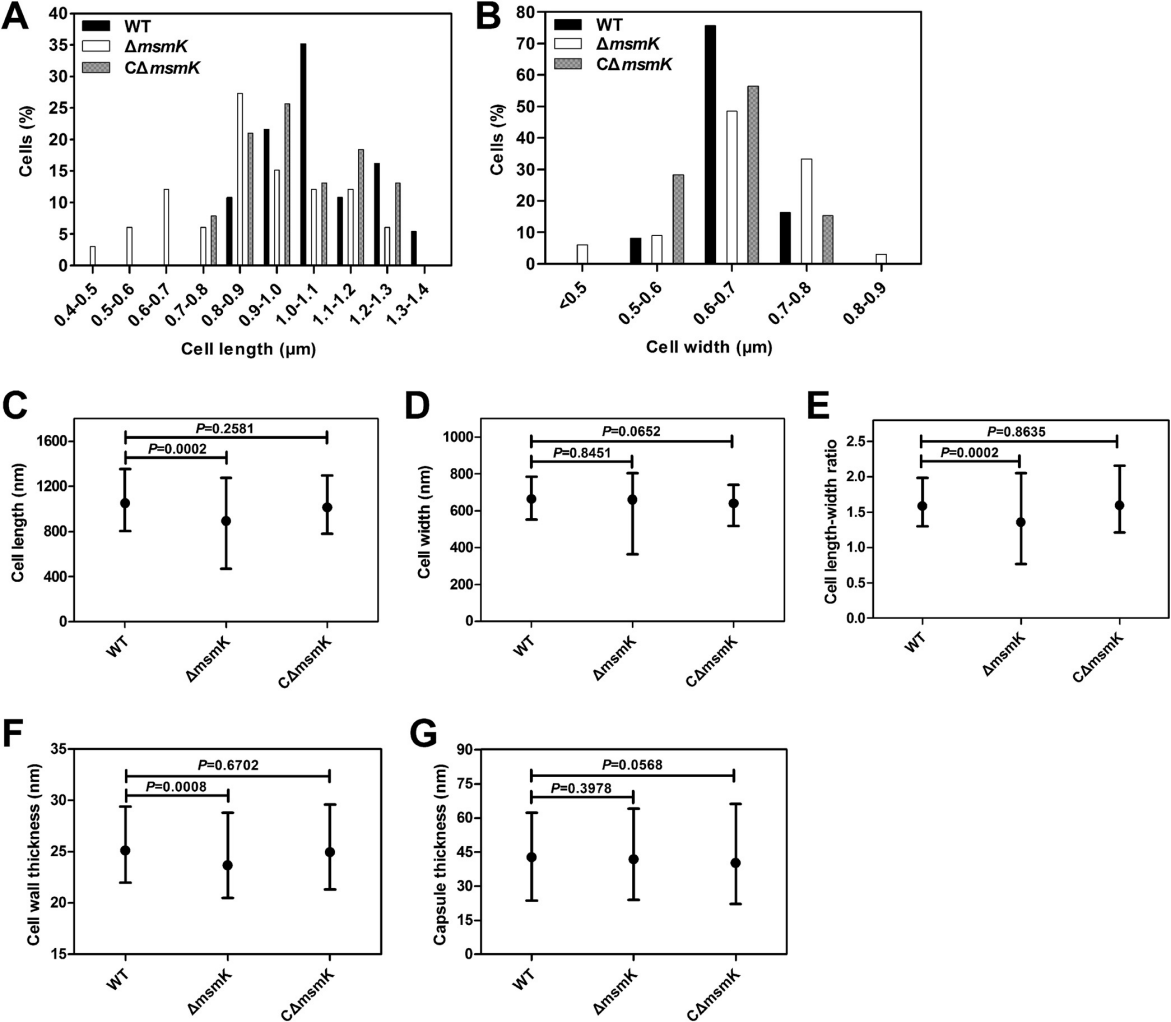
Comparative analysis of cell sizes. (A and B) Histogram statistics of cell length (A) and width (B) of the WT (black), Δ*msmK* (white), and CΔ*msmK* (gray) cells. (C to G) Cell size parameters of WT, Δ*msmK*, and CΔ*msmK* cells. Cell length (C), cell width (D), cell length-to-width ratio (E), cell wall thickness (F), and capsule thickness (G) are shown as means with ranges. Statistical analyses were performed via unpaired Student’s *t* tests in GraphPad Prism 6. Differences were considered significant on the basis of the calculated *P* values.

Electron micrographs revealed that WT cells appeared as characteristic elongated ellipsoids, and the bacteria lacking MsmK formed shorter cells than the WT cells (Fig. 6B). Cell longitudinal length (long axis) and cell width were measured using the automated Microbe-Tracker software (20). The distribution of distinct cell length classes differed between the WT and Δ*msmK* cells (Fig. 7A), and their cell width classifications did not substantially vary (Fig. 7B). The WT cells were concentrated in a cell length interval of 0.8 to 1.4 μm (*n *= 370), and the cells with lengths of 1.0 to 1.1 μm represented the highest proportion. The lengths of the Δ*msmK* cells were distributed in the range 0.4 to 1.3 μm (*n *= 352), and the cells with lengths of 0.8 to 0.9 μm represented the highest proportion. The proportions of WT cells with lengths of >1.0, 0.8 to 1.0, and <0.8 μm were 67.57% (250/370), 32.43% (120/370), and 0% (0/370), respectively. In comparison, the proportions of Δ*msmK* cells with lengths of >1.0, 0.8 to 1.0, and <0.8 μm were 30.68% (108/352), 42.33% (149/352), and 26.99% (95/352), respectively. On average, the cells lacking MsmK were significantly shorter (0.89 ± 0.20 μm) than the WT cells (1.05 ± 0.14 μm) (Fig. 7C). The widths of Δ*msmK* cells did not differ from those of WT cells (Fig. 7D). Therefore, the length-to-width ratio in mutants (1.35 ± 0.28) was significantly lower than that in WT cells (1.58 ± 0.21) (Fig. 7E). Calculated data for the three S. suis strains revealed that the cell wall in Δ*msmK* cells (25.14 ± 1.65 nm; *n *= 352) was also significantly thinner than that in WT cells (23.70 ± 1.95 nm; *n *= 370) (Fig. 7F). No difference in capsule thickness was observed among the three strains (Fig. 7G).

Although MsmK deletion resulted in the formation of chains and nearly spherical cells, the Δ*msmK* cells still displayed symmetrical cell division sites and equal-sized daughter cells either in chains or in diplococcal morphology (Fig. 6B). Division occurs in successive parallel planes that are perpendicular to their cell long axis, and small round cells continuously dividing with clearly visible division invagination rings were frequently observed (Fig. 6B). Consequently, the absence of MsmK did not disturb proper newborn septum placement and septal cell wall construction, thereby maintaining the capability of mutant cells to effectively divide.

### *msmK* complementation restores the mutant phenotype.

The recombinant plasmid with the *msmK* expression cassette under the control of its native promoter was successfully expressed in the mutants (Fig. 5B), and CΔ*msmK* cells showed phenotype characteristics similar to those of the WT cells (Fig. 6B). Analysis and comparison of cell sizes revealed that the lengths of CΔ*msmK* cells ranged 0.8 to 1.3 μm (*n *= 380) with an average value of 0.98 ± 0.15 μm (Fig. 7C). On average, the cell wall thickness of CΔ*msmK* cells was 24.96 ± 0.15 nm (*n *= 380). Therefore, *msmK* complementation fully restored the mutant morphology and size, and the above defective phenotypes of the Δ*msmK* cells were caused by *msmK* deletion. In summary, lack of MsmK leads to cell rounding, suggesting that this protein is involved in the maintenance of S. suis cell shape.

### MsmK absence affects peripheral PG synthesis.

**FIG 8:**
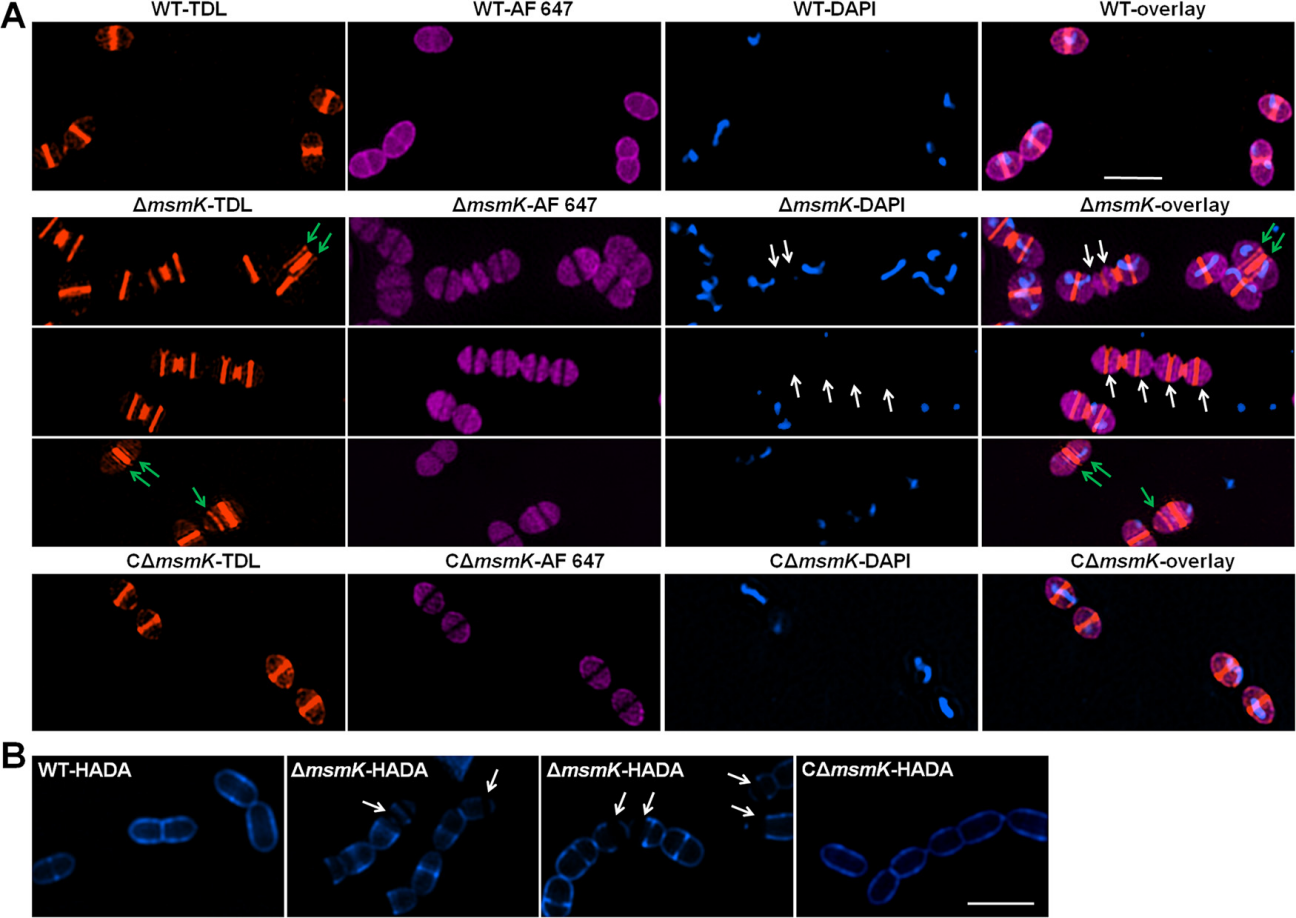
Observation of PG of the S. suis strains. (A) Colocalization of PG, membranes, and nucleoids. PG synthesis visualized through the incorporation of fluorescent d-amino acid derivatives (TDL; orange). Cells in log phase were grown in the presence of TDL to label nascent PG. Alexa Fluor 647 (AF 647; violet) was used to label cell membranes. Nucleoids were visualized with DAPI (blue). Overlays indicate the colocalization of cell walls, cell membranes, and nucleoids in the indicated cells. The green arrows indicate abnormal nascent PG walls in the mother cells that are distributed as parallel lines near the parental PG walls. The white arrows indicate cells lacking nucleoids caused by the narrow space between novel constructed cell walls. Bar, 2 μm. (B) Observation of peripheral PG synthesis. Cells in log phase were grown in the presence of HADA (blue) for one or two generations to label the cell contour. White arrows indicate PG older than one or two generations that could not be labeled. Bar, 2 μm.

Peripheral cell wall synthesis occurs between the future equator and newborn septum of dividing cells (18). Blocking this process through the deletion of peripheral growth components, such as PBP2b (18) and MreCD (21) in S. pneumoniae, produces similar-looking chains of nearly spherical cells. The decreased length-to-width ratio in Δ*msmK* cells suggests that MsmK might be involved in peripheral cell wall synthesis. Here, the morphology of nascent biosynthesized PG was examined by using TAMRA-d-lysine (TDL; a fluorescent carboxytetramethylrhodamine derivative of d-alanine) (22). S. suis strains were simultaneously labeled with TDL, membrane dyes, and 4′,6-diamidino-2-phenylindole (DAPI) and subjected to structured illumination microscopy to achieve colocalization. Superresolution micrographs of the WT cells displayed a typical pattern, with cell wall synthesis labeled at the midcell and equators of future daughter cells (Fig. 8A). As expected for the disruption of peripheral PG synthesis, the septal PG walls of 30.84% (70/227) of the mutant cells were constructed in mother cells without cell elongation (Fig. 8A), thereby generating two nascent equatorial rings that were extremely close to the parental division septum. Approximately 16.74% (38/227) of the mutant cells lacked nucleoids (Fig. 8A), and minicells (cell length of approximately 0.31 μm) with no nucleoid were observed. Moreover, part of the residual structure of PG did not fully disintegrate, resulting in incomplete cell separation.

S. suis strains were grown in the presence of HCC-amino-d-alanine (HADA), a fluorescent hydroxycoumarin derivative of d-alanine (22), for one or two generations to label the cell contour and further validate the effect of the absence of MsmK on peripheral PG synthesis. Compared with WT and CΔ*msmK* cells, part of the mutant cells displayed visualized septal walls and poles but an invisible peripheral cell wall (Fig. 8B). The missing blue contours in the Δ*msmK* cells represent the unlabeled older cell wall, in which new PGs were not inserted into the corresponding area at least in one or two generations. Therefore, MsmK is a component of the elongation system of S. suis.

### MsmK is not the phosphorylation target of STKs.

Eukaryotic-type serine/threonine kinases (STKs) are control elements in ovococci that coordinate several key processes over the cell cycle (5). The S. suis genome encodes a single STK with autophosphorylation and substrate phosphorylation activities and has several cell division-related targets, including FtsZ, FtsA, GpsB, DivIVA, and MapZ (23). After incubation with StkP and [γ-^32^P]ATP *in vitro*, no phosphorylation signal was observed on MsmK or its fragments (Fig. S3), thereby indicating that MsmK could not be phosphorylated by STK.

10.1128/mSphere.00119-21.6 FIG S3 Kinase assay *in vitro*. Purified MsmK and its fragments were subjected to phosphorylation by kinase STK in the presence of [γ-^32^P]ATP. Samples were resolved by SDS-PAGE, stained with Coomassie blue, and exposed to a sensitive screen. The image on the right shows the radioactive signal. Myelin basic protein (MBP) was used as a positive control to test the kinase activity of STK. Download FIG S3, TIF file, 1.3 MB. Copyright © 2021 Tan et al. 2021 Tan et al. https://creativecommons.org/licenses/by/4.0/ This content is distributed under the terms of the Creative Commons Attribution 4.0 International license.

## DISCUSSION

Faithful cell proliferation relies on the strict coordination of related cell division processes (3). To date, more than 30 proteins are proven or likely to function in the divisome and/or the elongation machineries in pneumococcal growth and division (3). Here, we identified a new cell division protein, MsmK, an ATPase that provides energy for multiple-carbohydrate transport in S. suis (15). The absence of MsmK in cells is not lethal but results in more chains and shorter daughter cells, some of which are anuclear, compared with the WT strain. These abnormal cells still possess symmetrical morphology postsplitting, indicating that the lack of MsmK does not affect the division site selection, whereas the absence of DivIVA (24), LocZ (20), or EzrA (25) causes cells to divide asymmetrically in ovococci. In addition, the absence of MsmK does not influence septal wall synthesis and cell division, except for the decreased separation degree between daughter cells.

The two-state model of PG synthesis in ovococci indicates the possibilities of ovococcus-to-rod or ovococcus-to-coccus transitions when septal and peripheral PG syntheses are separately inhibited (1). This hypothesis has been tested and approved by functional observations in several species, such as S. pneumoniae, Lactococcus lactis, Streptococcus agalactiae, Streptococcus bovis, and Enterococcus hirae (1). Conversely, related proteins could also be assigned to septal or peripheral PG synthesis based on cell morphology defects caused by deletion (18). Proteins with different functions, such as MreCD, RodA, RodZ, PBP1a, and PBP2b (4), are assigned to the elongation machinery of ovococci, and their deletions result in the same round phenotype. With regard to the production of chains and spherical cells caused by the absence of MsmK, we speculated that MsmK is involved in peripheral but not in septal wall synthesis. Subsequent electron and superresolution micrographs supported this hypothesis.

The function of MsmK in peripheral PG wall synthesis remains unclear. MsmK directly interacts with FtsZ *in vitro* and colocalizes with FtsZ *in vivo*. FtsZ has been reported to coordinate and organize not only septal PG synthesis but also peripheral PG synthesis in ovococci, similarly to the role of MreB in preseptal elongation in rods (1). Therefore, MsmK may affect peripheral PG synthesis by interacting with FtsZ and/or regulating FtsZ polymerization.

The mechanisms underlying the role of MsmK in the peripheral growth may be inspired by the conserved FtsEX complex. The FtsE/X complex is shown to be involved in activating cell wall hydrolases that impact the PG remodeling during cellular elongation in a variety of Gram-positive species (1). The membrane-bound FtsX interacts with the cytoplasmic FtsE ATPase subunit, structurally resembling an ABC transporter (26). The extracellular protein PcsB is the only essential PG autolysin (27), and the FtsEX complex is required for the PcsB function. The PG remodeling activity by PcsB is coordinated with cell division through its interaction with the FtsEX complex, which in turn cooperates with FtsZ and other division proteins through FtsE in S. pneumoniae (26). Notably, S. suis possesses the coding sequences of FtsE (SSUSC84_1271) and FtsX (SSUSC84_1270). Moreover, the chain phenotype of the mutant suggests that the PcsB function is affected in the absence of MsmK. As the ATPase subunit in S. suis (15) and S. pneumoniae (28), MsmK energizes multiple ABC transporters. Hence, MsmK may play the same bridge role as FtsE by linking FtsZ and specific functional proteins involved in the elongation system. The relationship between MsmK, FtsZ, FtsE/FtsX, PcsB, and other components involved in ABC transporter systems containing MsmK needs further study.

A number of proteins, such as CvaB of E. coli (29), SpoIVA of B. subtilis (30), Rep of beak and feather disease virus (31), and NS4B of classical swine fever virus (32), act as nucleoside triphosphatases (NTPases) that hydrolyze ATP and GTP with various levels of activity. Their ATPase and GTPase activities are associated with cell signaling, spore developing, viral replication, or membrane fusion. FeoB, a large cytoplasmic-membrane-associated protein of the ferrous iron transporter system, exhibits GTPase and ATPase activities in Vibrio cholerae and Helicobacter pylori but is solely a GTPase in E. coli (33). Here, we found that MsmK can display ATPase and GTPase activities. TEM and ultracentrifugation experiments showed that MsmK promotes FtsZ protofilament bundling in the GTP-dependent manner, and the addition of ATP decreases this promoting function. Considering that MsmK correspondingly forms distinct polymers in the presence of GTP or ATP, we speculate that the polymerized structure of MsmK relying on its GTPase activity is critical for its function in FtsZ polymerization. In this case, the dual enzyme activities of MsmK are responsible for its different functions. In addition, a series of proteins, such as ZapC (34), ZapA (35), and MciZ (36), promotes polymer synthesis by inhibiting the GTPase activity of FtsZ. Thus, MsmK might also competitively combine with GTP and disturb the GTPase activity of FtsZ, thereby promoting FtsZ polymerization.

MsmK interacts with FtsZ via its N terminus. However, the N-terminal fragment of MsmK showed lower-affinity interaction with FtsZ than full-length MsmK. The N-terminal fragment also showed low efficiency in promoting FtsZ polymerization. These results illustrated that the C-terminal region is important for the functions of MsmK. Walker A and B motifs contribute to the ATPase and GTPase activities of an NTPase, and mutations in the A motif have a relatively remarkable effect on the NTPase activity (31, 32). This phenomenon was also observed through the TEM analysis of A_MsmK and B_MsmK. Although the two protein mutants showed strong affinity for FtsZ, they inhibited FtsZ polymerization *in vitro*. That may be because they were unable to form appropriate structures due to the impaired GTPase activity, thereby severely affecting the formation and bundling of FtsZ protofilaments.

The E1α subunit of pyruvate dehydrogenase is required for the metabolism of pyruvate at the final stage of glycolysis and positively regulates Z-ring assembly by colocalizing with the chromosome in a pyruvate-dependent manner (37). Gluconate 5-dehydrogenase, which catalyzes the reversible reduction of 5-ketogluconate to d-gluconate, is localized to the cell division site and prevents ectopic Z-ring formation during cell division in S. suis (14). These findings clarified that nutritional conditions coordinate with bacterial division to ensure the survival of newly formed cells (38). We propose that MsmK is a nonessential component of cell division. MsmK is not a phosphorylated substrate of STK and thus is not regulated by STK. As a component of the carbohydrate ABC transporter, MsmK expression is naturally regulated by the nutrient level (39), suggesting that MsmK may be involved in coordinating bacterial division with nutrient availability. Whether MsmK serves as a linker between nutrition and cell division needs further study. Finally, MsmK is a nonunique protein that exists in numerous bacteria and is conserved independently of phylogenetic morphogenesis, thereby implying that its participation in cell division may be a widespread and fundamental physiological process in bacteria. Further developments in this area will ultimately help us understand, at a systems biology level, how bacteria can faithfully and continually multiply in a constantly changing environment.

## MATERIALS AND METHODS

### Bacterial strains, plasmids, and growth conditions.

Bacterial strains and plasmids are listed in Table S1. The WT strain (SC19, S. suis serotype 2) was isolated from a sick pig during an epidemic outbreak of S. suis infections in Sichuan Province, China (17). The *msmK* gene of SC19 was deleted via a double-crossover method through homologous recombination to generate a Δ*msmK* strain (15). For the construction of complemented strain CΔ*msmK*, the recombinant vector P*_msmK_*-*msmK* was transformed into Δ*msmK* cells (15). The construction of other S. suis strains is detailed in Text S1. All S. suis strains were grown at 37°C in tryptone soy broth (TSB; Difco, France) with 5% (vol/vol) fetal bovine serum (Sijiqing, Hangzhou, China). E. coli strains were grown in lysogeny broth (Difco) at 37°C. When necessary, the following antibiotics were added, as appropriate: erythromycin (90 μg/ml), streptomycin (20 μg/ml), spectinomycin (100 μg/ml), kanamycin (25 μg/ml), chloromycetin (25 μg/ml), and tetracycline (12.5 μg/ml). Unless otherwise specified, all antibiotics, chemicals, and substrates were purchased from Biosharp (Hefei, China).

10.1128/mSphere.00119-21.1 TABLE S1 Bacterial strains and plasmids used in this study. Download Table S1, DOC file, 0.07 MB. Copyright © 2021 Tan et al. 2021 Tan et al. https://creativecommons.org/licenses/by/4.0/ This content is distributed under the terms of the Creative Commons Attribution 4.0 International license.

10.1128/mSphere.00119-21.3 TEXT S1 Supplemental materials and methods. Download Text S1, DOC file, 0.10 MB. Copyright © 2021 Tan et al. 2021 Tan et al. https://creativecommons.org/licenses/by/4.0/ This content is distributed under the terms of the Creative Commons Attribution 4.0 International license.

### General methods.

Oligonucleotides used are listed in Table S2 in the supplemental material. Cloning and genetic manipulations were conducted using standard techniques. Recombinant plasmids and strains, protein techniques, general immunoblotting assays, bacterial two-hybrid and SPR analyses, and GTPase, pelleting, and kinase assays were performed as described in Text S1.

10.1128/mSphere.00119-21.2 TABLE S2 Primers used in this work. Download Table S2, DOC file, 0.05 MB. Copyright © 2021 Tan et al. 2021 Tan et al. https://creativecommons.org/licenses/by/4.0/ This content is distributed under the terms of the Creative Commons Attribution 4.0 International license.

### Coimmunoprecipitation.

*In vivo* interaction assays were performed as described previously (40) with few modifications. Cultures of the CΔ*msmK* and CΔ*msmK*-His strains were grown exponentially in TSB to an OD_600_ of ∼0.6 to 0.8. Cells were collected, washed, and resuspended with 1× phosphate-buffered saline (PBS) buffer at 4°C. A 10% paraformaldehyde solution was added for cross-linking to a final concentration of 0.1% (vol/vol). Mixtures were incubated at 37°C for 1 h. Exactly 1.0 M glycine was added and incubated at 25°C for 10 min to abolish cross-linking reactions. The cells were then collected, washed, and resuspended with lysis buffer containing 50 mM Tris-HCl (pH 7.4), 150 mM NaCl, 5 mg/ml lysozyme, 1% Triton X-100 (vol/vol), and 1% phenylmethylsulfonyl fluoride (vol/vol). After sonication, bacterial debris was removed through centrifugation at 14,000 × *g* for 10 min at 4°C. The supernatant of each strain was incubated with Ni-NTA columns (GE Healthcare, USA) at 4°C for 2 h in binding buffer containing 50 mM Tris-HCl (pH 7.4), 150 mM NaCl, and 25 mM imidazole. The beads were washed four times with 80 mM imidazole for 5 min each. His-tagged protein was eluted by incubating with 250 mM imidazole for 1.5 h at 4°C. The original supernatant (input) and the final elution of each strain were separated by polyacrylamide gel electrophoresis and transferred to a polyvinylidene fluoride membrane (Invitrogen). The membrane was then probed with mouse antihistidase antibody (1:4,000; Abcam, China), in-house-prepared anti-MsmK serum (1:1,000), or in-house-prepared anti-FtsZ serum (1:1,000). This experiment was performed in triplicate.

### Electron microscopy.

All purified proteins were precentrifuged for 10 min at 16,000 × *g* at 4°C. Proteins (0.6 μM each) were incubated with 2 mM GTP and/or 1 mM ATP for 10 min at 37°C in buffer P (Text S1) to observe the structure formation of MsmK and its derivatives. For observing FtsZ polymerization *in vitro*, mixtures of 0.6 μM FtsZ and/or His-tagged MsmK constructs (0.6 μM each) were incubated at 37°C for 2 min in buffer P to analyze FtsZ polymerization *in vitro*. Afterward, 2 mM GTP and/or 1 mM ATP was added into the mixtures, which were continuously incubated for 10 min at 37°C. Mixtures were then withdrawn and applied to glow-discharged carbon-coated grids. The grids were air-dried and visualized using an H-7650 TEM (Hitachi, Japan).

The cells in TSB were grown to an OD_600_ of 0.5 and fixed overnight with 2.5% glutaraldehyde at 4°C to observe the bacterial morphology under TEM. For SEM, the cell cultures were grown in TSB to an OD_600_ of 0.5 and spotted onto poly-l-lysine-coated coverslips, followed by washing with PBS buffer. The cells were fixed overnight with 2.5% glutaraldehyde at 4°C. Subsequent dehydration steps were performed using ethanol as previously described (19). The dried samples were covered with a 10-nm-thick layer of gold and observed with a JSM-6390LV SEM (JEOL, Japan).

### Fluorescence microscopy.

The immunofluorescence microscopy of cytoplasmic proteins was performed as previously described (14), with a few modifications. The CΔ*msmK*-GFP strain expressing the MsmK-GFP fusion protein was cultivated in TSB at 37°C until an OD_600_ of 0.6 was achieved. After washing three times with precooled PBS, cultures were incubated with 100% formaldehyde for 20 min at −20°C and with 80% formaldehyde for 1 h at 28°C. Cells were washed once with 80% methanol and permeabilized with 0.3% Triton X-100 in PBS for 30 min at 28°C. After blocking with 5% bovine serum albumin for 30 min at 37°C, cells were sequentially probed with the mouse anti-FtsZ serum (1:100) and the goat anti-mouse IgG (heavy plus light chain [H+L])-Cy3 conjugate (1:200; Proteintech Group. Inc., USA) for 1 h at 37°C. Cells were transferred onto microscope slides and covered by 1% agarose gel. Superresolution images were obtained using a Nikon structured illumination microscope (N-SIM; Nikon Instruments, Inc.) equipped with an electron-multiplying charge-coupled device camera (iXon DU-897; Andor) and a total internal reflection fluorescence objective (100×; numerical aperture [NA], 1.49; CFI Apo TIRF; Nikon Instruments, Inc.). Images were captured with multiple phases and angles of illumination pattern before direct reconstruction by using the Nikon NIS-Elements AR software.

TDL and HADA (22) were synthesized by Wuxi AppTec Co., Ltd. TDL or HADA was dissolved in dimethyl sulfoxide (up to 0.2 M) to probe peptidoglycan synthesis. Alexa Fluor 647 (AF 647; Thermo Scientific) dissolved in anhydrous acetonitrile (up to 10 mg/ml) was prepared for cell membrane staining. For the colocalization of PG cell walls, cell membranes, and nucleoids, cultures of WT, Δ*msmK*, and CΔ*msmK* strains were grown to an OD_600_ of 0.5 and then centrifuged at 9,000 × *g* for 5 min. After washing with PBS, the cells were resuspended in 1 ml of PBS containing 5 μl of TDL and 5 μl of AF 647. The mixtures were incubated for 1 h at 37°C in the dark. The cells were then washed with PBS and stained with 1.5 μg/ml DAPI for 5 min in the dark at room temperature. Following the same incubation and pretreatment steps, the cells were incubated in 1 ml of PBS containing 5 μl of HADA for 30 min at 37°C in the dark to observe the cell wall contours.

### Image analysis.

Cell sizes were measured with the automated MicrobeTracker software (20) by using TEM images from three independent experiments. GTPase assays and pelleting assays were quantitatively analyzed using ImageJ2x software. Statistical analyses were performed via unpaired Student’s *t* tests in GraphPad Prism 6.
