# Supplementary figures and images for: Streptococcus suis MsmK: Novel Cell Division Protein Interacting with FtsZ and Maintaining Cell Shape
Source: mSphere. 2021 Mar 17;6(2):e00119-21. doi: 10.1128/mSphere.00119-21 (PMC8546688; doi:10.1128/mSphere.00119-21)

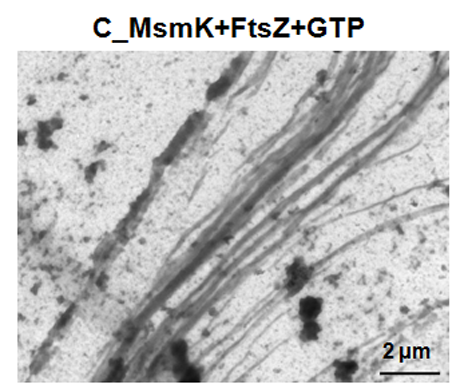

Supplement: FIG S2 [file msphere.00119-21-sf002.tif]

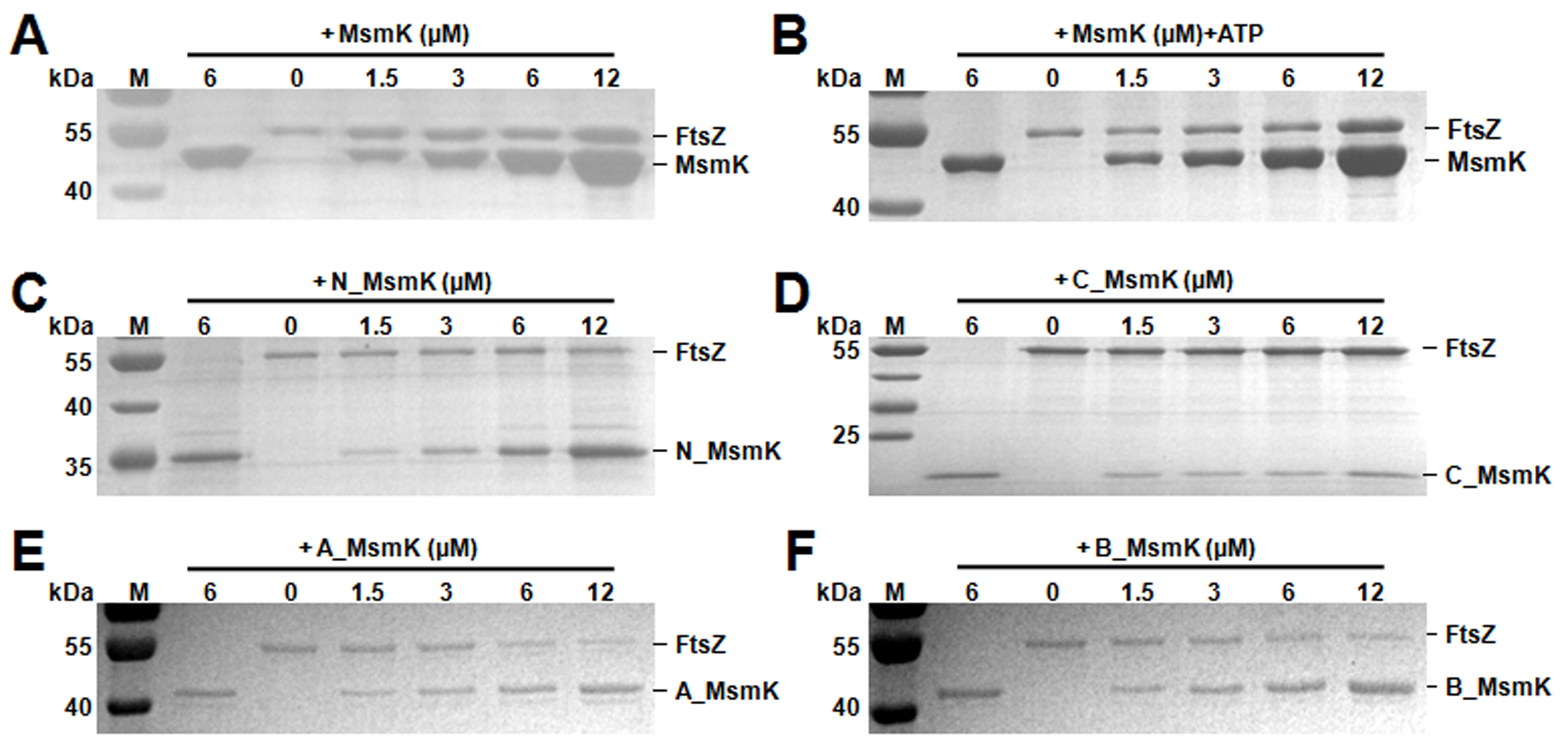

Supplement: FIG S1 [file msphere.00119-21-sf001.tif]

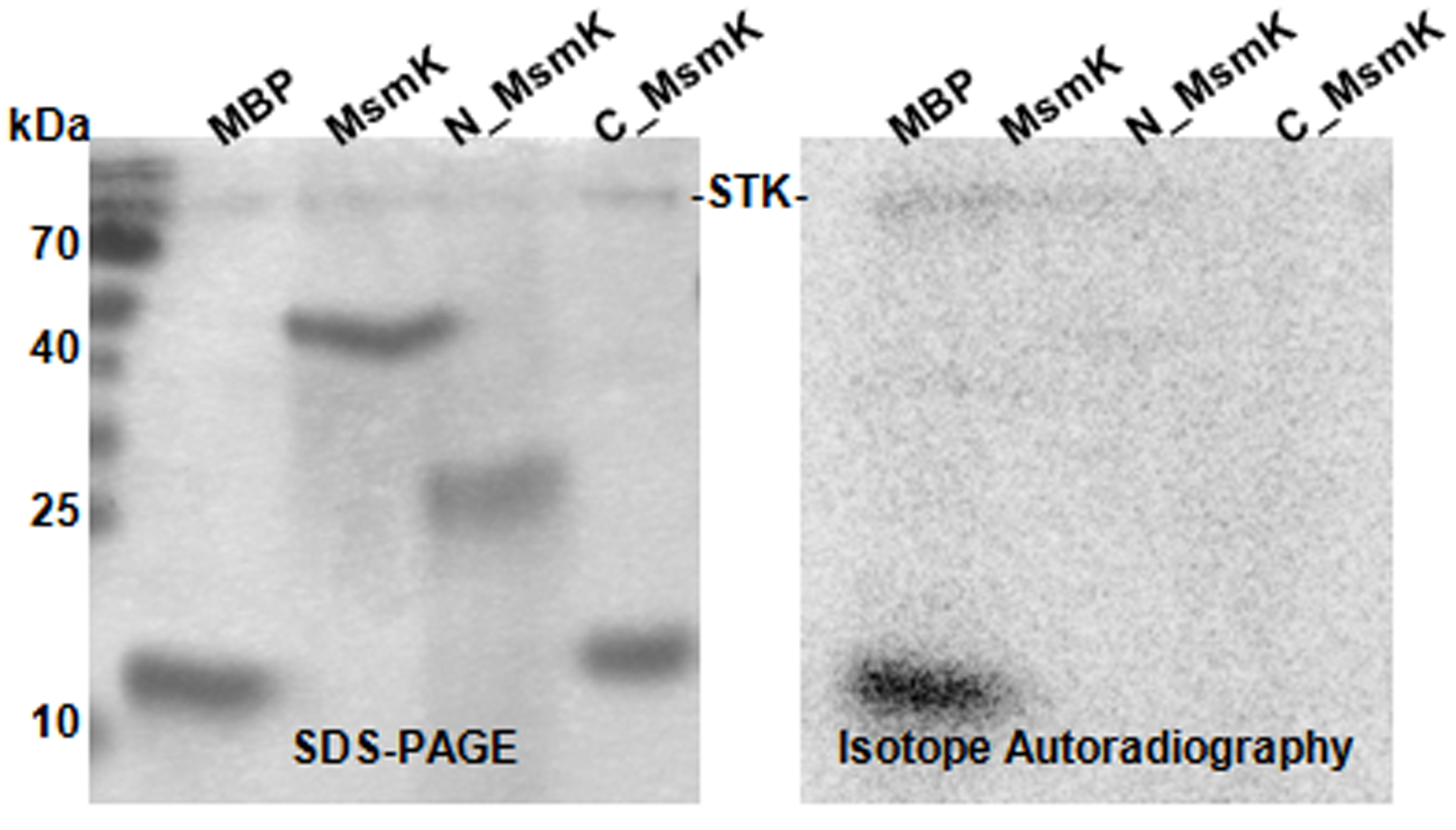

Supplement: FIG S3 [file msphere.00119-21-sf003.tif]
