# Supplementary material for: Streptococcus suis MsmK: Novel Cell Division Protein Interacting with FtsZ and Maintaining Cell Shape
Source: mSphere. 2021 Mar 17;6(2):e00119-21. doi: 10.1128/mSphere.00119-21 (PMC8546688; doi:10.1128/mSphere.00119-21)
Supplement: TABLE S1 [file msphere.00119-21-st001.doc]

**Table S1 Bacterial strains and plasmids used in this study**

| **Strains/plasmids** | **Characteristics*^a^*** | **Source** |
| --- | --- | --- |
| **Strains** |  |  |
| *S. suis* strains |  |  |
| SC19 | Virulent Chinese *S. suis* serotype 2 isolate, the wild-type | Laboratory storage |
| Δ*msmK* | SC19 *msmK*::*erm*; The *msmK* deletion mutant of strain SC19; Erm^r^ |  |
| CΔ*msmK* | SC19 Δ*msmK* *msmK*^+^; The complemented strain of Δ*msmK*; Erm^r^, Spc^r^ |  |
| CΔ*msmK*-His | SC19 Δ*msmK msmK*-*his*^+^; Erm^r^, Spc^r^ | This work |
| CΔ*msmK*-GFP | SC19 Δ*msmK msmK*-*gfp*^+^; Erm^r^, Spc^r^ | This work |
| *E. coli* strains |  |  |
| DH5α | Cloning host for maintaining recombinant plasmids | Trans |
| BL21 (DE3) | Expression host for exogenous protein production | Trans |
| XL1-Blue | Host Strain for propagating pBT and pTRG recombinants; Kan^r^ | Stratagene |
| **Plasmids** |  |  |
| pBT | Bait plasmid for bacterial two-hybrid system; Chl^r^ | Stratagene |
| pBT-LGF2 | Positive control plasmid; Chl^r^ | Stratagene |
| pBT-*ftsZ* | pBT with the full-length *ftsZ*; Chl^r^ | This work |
| pTRG | Target plasmid for bacterial two-hybrid system; Tet^r^ | Stratagene |
| pTRG-Gal | Positive control plasmid; Tet^r^ | Stratagene |
| pTRG-*msmK* | pTRG with the full-length *msmK*; Tet^r^ | This work |
| pET-28a/c | His tag fusion expression vectors; Kan^r^ | Novagen |
| pET-F | pET-28c with the full-length *ftsZ*; Kan^r^ | This work |
| pET-M | pET-28a with the full-length *msmK*; Kan^r^ | This work |
| pET-NM | pET-28a with the amino terminal domain of *msmK*; Kan^r^ | This work |
| pET-CM | pET-28a with the carboxyl terminal domain of *msmK*; Kan^r^ | This work |
| pET-AM | pET-28a with the walker A deletion of *msmK*; Kan^r^ | This work |
| pET-BM | pET-28a with the walker B deletion of *msmK*; Kan^r^ | This work |
| pET-1444 | pET-28a with the full-length SSUSC84_1444; Kan^r^ | This work |
| pSET2 | The *E. coli* DH5α-*S. suis* shuttle vector; Spc^r^ |  |
| P*_msmK_*-*msmK* | pSET2 with the expression cassette of MsmK; Spc^r^ |  |
| P*_msmK_*-*msmK*-*his* | pSET2 with the expression cassette of MsmK-His; Spc^r^ | This work |
| pMIDG310 | A plasmid containing the GFP coding sequence | (3) |
| P*_msmK_*-*msmK*-*gfp* | pSET2 with the expression cassette of MsmK-GFP; Spc^r^ | This work |

***^a^*** Erm^r^, erythromycin resistant; Spc^r^, spectinomycin resistant; Kan^r^, kanamycin resistant; Chl^r^, chloromycetin resistant; Tet^r^, tetracycline resistant.

**References**

1. Tan MF, Gao T, Liu WQ, Zhang CY, Yang X. 2015. MsmK, an ATPase, Contributes to Utilization of Multiple Carbohydrates and Host Colonization of *Streptococcus suis*. PloS one 10:e0130792. http://www.ncbi.nlm.nih.gov/pubmed/26222651.

2. Takamatsu D, Osaki M, Sekizaki T. 2001. Construction and characterization of *Streptococcus suis*-*Escherichia coli* shuttle cloning vectors. Plasmid 45:101-113. http://www.ncbi.nlm.nih.gov/pubmed/11322824.

3. Zhang T, Ding Y, Li T, Wan Y, Li W, Chen H, Zhou R. 2012. A Fur-like protein PerR regulates two oxidative stress response related operons dpr and metQIN in *Streptococcus suis*. BMC microbiology 12:85. http://www.ncbi.nlm.nih.gov/pubmed/22646062.
