# Supplementary material for: Streptococcus suis MsmK: Novel Cell Division Protein Interacting with FtsZ and Maintaining Cell Shape
Source: mSphere. 2021 Mar 17;6(2):e00119-21. doi: 10.1128/mSphere.00119-21 (PMC8546688; doi:10.1128/mSphere.00119-21)
Supplement: TEXT S1 [file msphere.00119-21-s0001.doc]

**SUPPLEMENTAL MATERIALS AND METHODS**

**Construction of plasmids.** For cloning and expression, the *ftsZ* gene was amplified by PCR from the *Streptococcus suis* SC19 chromosomal DNA by using primers *ftsZ*-F*/ftsZ*-R carrying the *EcoR*I/*Xho*I restriction enzyme sites. After digestion, the PCR product was inserted into the digested pBT and pET-28c vectors to generate the recombinant plasmids pBT-*ftsZ* and pET-F. Three pairs of specific primers, namely, *msmK*-F/*msmK*-R, N_*msmK*-F/N_*msmK*-R, and C_*msmK*-F/C_*msmK*-R, carrying *BamH*I and *Xho*I sites, were used to clone *msmK* regions from the SC19 genome. Digested DNA fragments were cloned to the digested pET-28a to generate the recombinant plasmids pET-M, pET-NM, and pET-CM. The digested full-length *msmK* was also cloned into the pTRG vector and generate the recombinant plasmid pTRG-*msmK*. Moreover, the coding sequence of SSUSC84_1444 was amplified using primers 1444-F*/*1444-R with the *Nco*I and *Xho*I restriction enzyme sites. After digestion, the PCR product was inserted into digested pET-28a to generate the recombinant plasmid pET-1444.

The NEBuilder® HiFi DNA assembly cloning kit was used to assemble the His-fused gene expression cassettes through homologous recombination. Primers mwA1-F/mwA1-R were used to amplify the former fragment of the *msmK* gene (1–117 base pairs) and construct the pET-AM. The primers mwA2-F/mwA2-R were used to amplify the latter fragment of the *msmK* gene (142–1137 base pairs). The two DNA fragments were mixed with linearized pET-28a (digested by *Nco*I and *Xho*I) on ice and incubated in a thermocycler at 50°C for 15 min. Then, 2 μL of the assembled product was transformed into competent DH5α cells following the transformation protocol. Positive recombinant plasmids were verified and confirmed by DNA sequencing in the Beijing Genomics Institute (BGI).

Primers mwA1-F/mwB1-R and mwB2-F/mwA2-R were used to amplify the former region of the *msmK* coding sequence (1–471 base pairs) and the latter region of the *msmK* coding sequence (487–1137 base pairs), respectively, to construct the pET-BM. Subsequent reorganization experiments were performed according to the transformation protocol. Positive recombinant plasmids were confirmed by DNA sequencing in the BGI.

The native promoter and *msmK* coding sequence were amplified with primers pmh1-F/pmh1-R from the SC19 genome to construct the recombinant plasmids P*_msmK_*-*msmK*-*his* and P*_msmK_*-*msmK*-*gfp*. *His* tag coding sequence was amplified with primers pmh2-F/pmh2-R using PET-28a as a template. The coding sequence of *gfp* gene was amplified using primers *gfp*-F*/gfp*-R from the plasmid pMIDG310. Two specific DNA fragment pairs were mixed with the linearized *S. suis*–*Escherichia coli* shuttle cloning vector pSET2 (digested by *EcoR*I and *BamH*I) and transformed into competent DH5α cells according to the transformation protocol. Positive recombinant plasmids were confirmed by DNA sequencing in the BGI.

**Construction of the *S. suis* strains.** Detailed information of mutagenesis and genetic complementation of *msmK* in SC19 is presented in the previous reference . The CΔ*msmK*-His and CΔ*msmK*-GFP strains used for expressing tagged MsmK under control of the native promoter of *msmK* (P*_msmK_*) were obtained by transforming the *msmK* mutant strain Δ*msmK* with P*_msmK_*-*msmK*-*his* and P*_msmK_*-*msmK*-*gfp*, separately. Positive transformants were selected using spectinomycin as a marker.

**Protein expression and purification.** All recombinant pET-28a/c vectors were expressed with an N-terminal 6× His-tag in *E. coli* BL21 (DE3) by induction with 1 mM of isopropyl-β-D-thiogalactopyranoside overnight at 18°C for 12 h. His-tagged proteins were purified by using Ni-NTA columns (GE Healthcare) under the natural condition according to the manufacturer’s recommendation. The purified protein was desalted in phosphate-buffered saline (PBS) buffer with centrifugal filters (Millipore). The quality and quantity of purified proteins were tested by sodium dodecyl sulfate-polyacrylamide gel electrophoresis (SDS-PAGE) and a Micro BCA protein assay kit (Cwbiotech), respectively. Finally, the purified protein was stored at −80°C.

**Immunoblot analysis.** Purified his-tagged proteins were separated on 12% SDS-PAGE gels and transferred to polyvinylidene fluoride membranes (Invitrogen) by using a semi-dry blotting system (Bio-rad). After blocking in 5% skim milk in TBST buffer (20 mM Tris/HCl, pH 8.0, 150 mM NaCl, and 0.05% Tween-20), the membranes were incubated with mouse anti-histidase antibody (1:4,000; Abcam) at 37°C for 2 h. Subsequently, goat anti-mouse IgG (H+L)-HRP (1:5,000; Abcam) was added and incubated at 37°C for 1 h. Detection was conducted by using a Western ECL substrate kit (Bio-Rad). Membranes were viewed using an ECL plus Western Blot detection system (DNR).

Far-western blot analysis was performed as previously described . MsmK and its derivatives were separated on 12% SDS-PAGE gel and then electrotransferred to a polyvinylidene fluoride membrane. The membrane was blocked in 5% skim milk in TBST buffer and then incubated in 10 mg/mL His-tagged FtsZ at 4°C overnight. After stringent washing, the membrane was probed with homemade mouse anti-FtsZ polyclonal serum (1:1,000) at 37°C for 2 h and then incubated with goat anti-mouse IgG (H+L)-HRP. SSUSC84_1444, an ATPase that belongs to ATP-binding cassette-type glycine betaine transport system and has the highest identity (40.40%) to MsmK among its homologs in *S. suis*. Here, the His-tagged protein of SSUSC84_1444 served as the negative control. This assay was repeated three times.

WT, Δ*msmK*, or CΔ*msmK* cells were cultured in tryptone soya broth to analyze the protein expression levels of MsmK and FtsZ. Cultures in exponential phase were collected through centrifugation at 12,000 ×*g* for 10 min and resuspended in bacterial lysis buffer (50 mM Tris/HCl, pH 8.5, 100 mM NaCl, 2 mM EDTA, 1 mM phenylmethanesulfonyl fluoride, 100 μg/mL of lysozyme, and 0.5% Triton X-100). The cells were then lysed with a French pressure cell press, and the supernatant was preserved for Western blot analysis by centrifugation at 12,000 ×*g* at 4°C for 30 min. Quantified bacterial lysate was separated by SDS-PAGE and was probed at 37°C for 2 h with 1:1,000 diluted homemade mouse anti-MsmK serum , mouse anti-FtsZ serum, or mouse anti-Enolase serum. Enolase , a glycolytic enzyme that catalyzes the conversion of phosphoglycerate to phosphoenolpyruvate of *S. suis*, was used as the internal reference due to its stable expression levels outside the host.

**Bacterial two-hybrid analysis.** The BacterioMatch®II Two-Hybrid System (Stratagene) was used to establish protein–protein interactions in accordance with the manufacturer’s recommendations. The pBT and pTRG vectors containing *ftsZ* and *msmK* were generated separately. A series of pBT- and pTRG-related plasmids was co-transformed into the reporter *E. coli* strain XL1-Blue. Lysogeny broth medium plates containing 12.5 mg/mL tetracycline and 25 mg/mL chloramphenicol were used to select positive co-transformants. The selective medium contained 5 mM 3-amino-1,2,4-triazole (3-AT), 12.5 mg/mL streptomycin, 12.5 mg/mL tetracycline, 25 mg/mL chloramphenicol, and 50 mg/mL kanamycin. The nonselective medium was generated without 3-AT and streptomycin. The liquid culture (10 μL, an OD_600_ of 0.5) of each co-transformant was spotted onto the selective and nonselective plates, which were then incubated at 37°C for 2–3 days in the dark. The co-transformant containing pBT-LGF2 and pTRG-Gal11P was used as a positive control for expected growth on the selective plates. A co-transformant containing the empty vectors pBT and pTRG-*msmK* was employed as a self-activation control. A co-transformant containing the empty vectors pBT and pTRG was applied as a negative control. Colonies that can grow on selective plates were considered to possess positive protein–protein interactions.

**Surface plasmon resonance analysis.** Surface plasmon resonance analysis was performed by using the ProteOn XPR36 protein interaction array system (Bio-rad). His-tag fused FtsZ was immobilized on the ProteOn GLC sensor chip with final immobilization levels of ~ 3,000 resonance units (RU, 1 RU = 1 pg protein/mm^2^) in 10 mM sodium acetate buffer (pH 4.0) for all SPR assays. The system was equilibrated with PBS-T buffer (pH 7.4, 10 mM sodium phosphate, 150 mM NaCl, 0.05% Tween 20). After the 90° rotation of the fluid system, his-tag fused MsmK protein constructs in PBS-T buffer were injected in parallel flow channels. The PBS-T buffer without protein was simultaneously injected in the last channel as the negative reference. Resulting analysis was fitted by the simplest 1:1 interaction model to obtain the corresponding equilibrium association and dissociation constants (*K*_a_ and *K*_d_). All SPR assays were performed at 25°C and repeated at least three times.

**GTPase assays.** GTP hydrolysis by the MsmK constructs were assayed as described previously . Purified proteins at the indicated concentrations were preincubated at 30°C for 10 min in buffer P. The reaction was started by adding [α-^32^P]GTP (0.33 Ci/mol; PerkinElmer) up to a final concentration of 2 mM. When necessary, 1 mM [α-^32^P]ATP (3 Ci/mol; Perkin Elmer) was used. After incubation at 30°C for 1 h, 2.5 µL of the samples were obtained and transferred onto TLC PEI-cellulose F plastic sheets (Merck). The sheets were developed in a solvent system containing 1 M LiCl and 0.5 M formic acid in H_2_O, air-dried, and exposed to a phosphor screen (GE Healthcare). The screen was scanned using a phosphor imaging analysis system (Fujifilm). Bovine serum albumin was used as the negative control. Each assay was repeated three times.

**Pelleting assays.** The assays were conducted as previously described with some modifications. Mixtures of 6 μM FtsZ and/or his-tagged MsmK constructs at the indicated concentrations were incubated for 2 min at 37°C in buffer P (50 mM Hepes/NaOH, pH 7.2, 50 mM KCl, 10 mM MgCl_2_, 1 mM β-mercaptoethanol). Then 2 mM GTP and/or 1 mM ATP was added into the mixtures and incubated for 10 min at 37°C. The solutions were subsequently centrifuged for 20 min at 254,000 ×*g* and 25°C with a Beckman TL-100 ultracentrifuge. Pelletized proteins were dissolved and subjected to electrophoresis. The gels were scanned and collected with the Gel Doc^TM^ XR+ imager. Each assay was repeated three times.

**Kinase assays.** *In vitro* kinase assay was performed as previously described . The reaction was conducted in 50 μL of kinase buffer (50 mM Hepes, 1 mM dithiothreitol, 0.01% Brij-35, and pH 7.0) containing 2 mM MnCl_2_, 100 μM ATP, and 1 μCi [γ-^32^P]ATP (3,000 Ci/mmol; Perkin Elmer). The enzyme/substrate ratio was 1:10 with 0.4 μM kinase. StkP and the MsmK constructs were incubated in the kinase buffer for 30 min at 37°C and stopped by adding SDS-PAGE sample buffer with EDTA (25 mM final). Proteins were then separated in 12% SDS-PAGE gel and exposed to the phosphor screen. Subsequently, the gels were stained with Commassie brilliant blue and the screen was scanned using the phosphor imaging analysis system. Radioactive samples were analyzed by using a PhosphorImager apparatus (Fujifilm) to obtain the relative quantification of the radio-labelled GDP incorporation. Myelin basic protein (Sigma) was used as a positive control to test kinase activity.

**References**

1. Tan MF, Gao T, Liu WQ, Zhang CY, Yang X. 2015. MsmK, an ATPase, Contributes to Utilization of Multiple Carbohydrates and Host Colonization of *Streptococcus suis*. PloS one 10:e0130792.

2. Li W, Wan Y, Tao Z, Chen H, Zhou R. 2013. A novel fibronectin-binding protein of *Streptococcus suis* serotype 2 contributes to epithelial cell invasion and in vivo dissemination. Veterinary microbiology 162:186-194.

3. Zhang A, Chen B, Mu X, Li R, Zheng P, Zhao Y, Chen H, Jin M. 2009. Identification and characterization of a novel protective antigen, Enolase of *Streptococcus suis* serotype 2. Vaccine 27:1348-1353.

4. Thanbichler M, Shapiro L. 2006. MipZ, a spatial regulator coordinating chromosome segregation with cell division in Caulobacter. Cell 126:147-162.

5. Giefing C, Jelencsics KE, Gelbmann D, Senn BM, Nagy E. 2010. The pneumococcal eukaryotic-type serine/threonine protein kinase StkP co-localizes with the cell division apparatus and interacts with FtsZ *in vitro*. Microbiology 156:1697-1707.

6. Zhang C, Sun W, Tan M, Dong M, Liu W, Gao T, Li L, Xu Z, Zhou R. 2017. The Eukaryote-Like Serine/Threonine Kinase STK Regulates the Growth and Metabolism of Zoonotic *Streptococcus suis*. Frontiers in cellular and infection microbiology 7:66.
