# Supplementary material for: Streptococcus suis MsmK: Novel Cell Division Protein Interacting with FtsZ and Maintaining Cell Shape
Source: mSphere. 2021 Mar 17;6(2):e00119-21. doi: 10.1128/mSphere.00119-21 (PMC8546688; doi:10.1128/mSphere.00119-21)
Supplement: TABLE S2 [file msphere.00119-21-st002.doc]

**Table S2** Primers used in this work

| **Primers** | **Sequence (5ʹ-3ʹ) *^a^*** | **target** |
| --- | --- | --- |
| **General PCR amplification** |  |  |
| *ftsZ*-F (*EcoR*I) | cggaattccgatggcattttcatttgaagca | *ftsZ* coding sequence |
| *ftsZ*-R (*Xho*I) | cgctcgagttagcgattacggaagaatg |
| *msmK*-F (*BamH*I) | cgggatccttgaacatggttcaattgaatt | *msmK* coding sequence |
| *msmK*-R (*Xho*I) | cgctcgagttagacaatcgccttgcttg |
| N_*msmK*-F (*BamH*I) | cgggatccttgaacatggttcaattgaatt | the amino terminal of *msmK* |
| N_*msmK*-R (*Xho*I) | cgctcgagttactcttctgggctaccgat |
| C_*msmK*-F (*BamH*I) | cgggatccatgctctacaaccgccctgtt | the carboxyl terminal of *msmK* |
| C_*msmK*-R (*Xho*I) | cgctcgagttagacaatcgccttgcttg |
| 1444-F (*Nco*I) | GCGCCATGGatgatgattgattatcaaaat | SSUSC84_1444 coding sequence |
| 1444-R (*Xho*I) | cgctcgagCACCACCACCACCACCACT |
| **Homologous recombination** |  |  |
| **For pET-AM** |  |  |
| mwA1-F | TTTAAGAAGGAGATATACCttgaacatggttcaattgaatt | the former fragment of *m*smK (1-117 base pairs) |
| mwA1-R | CAATCATACGAAGAGTTGTTACAAATACGATAAACTCC |
| mwA2-F | ggagtttatcgtatttgtaacaactcttcgtatgattg | the latter fragment of *msmK* (142-1137 base pairs) |
| mwA2-R | AGTGGTGGTGGTGGTGGTGGACAATCGCCTTGCTTGTTT |
| **For pET-BM** |  |  |
| mwB1-R | TCCAAGTTTGACAAAGGTTCTTTTGCATCACGAACGATGG | the former fragment of *m*smK (1-471 base pairs) |
| mwB2-F | ccatcgttcgtgatgcaaaagaacctttgtcaaacttgga | the latter fragment of *msmK* (487-1137 base pairs) |
| **For P*_msmK_*-*msmK*-*his*** |  |  |
| pmh1-F | GCCTGCAGGTCGACTCTAGATAGGAGCTAATGATGAGAAAA | the native promoter and *msmK* coding sequence |
| pmh1-R | CTGCAGGAACTCGATGTCTAGTTTGACAATCGCCTTGCTTGTTTC |
| pmh2-F | GTGATGATGATGATGATGATGATGCTGCAGGAACTCGATGTCTAG | *his* tag coding sequence |
| pmh2-R | GTTGTAAAACGACGGCCAGTTTAGTGATGATGATGATGATGATGA |
| **For P*_msmK_*-*msmK*-*gfp*** |  |  |
| *gfp*-F | AAACTAGACATCGAGTTCCTGCAGATGAGTAAAGGAGAAGAAC | *gfp* coding sequence |
| *gfp*-R | TTGTAAAACGACGGCCAGTCTATTTGTATAGTTCATCC |

***^a^*** Underlined sequences represent the restriction sites. All primers were designed in this work.
